# *Mentha rotundifolia*, a Source of Amoebicidal Agents Against *Naegleria fowleri*

**DOI:** 10.3390/ijms26189048

**Published:** 2025-09-17

**Authors:** Meriam Ben Youssef, Javier Chao-Pellicer, Eduardo Hernández-Álvarez, Amani Omrani, Ines Sifaoui, Hichem Sebai, Isabel L. Bazzocchi, José E. Piñero, Ignacio A. Jiménez, Jacob Lorenzo-Morales

**Affiliations:** 1 Instituto Universitario de Enfermedades Tropicales y Salud Pública de Canarias, Universidad de La Laguna, 38296 La Laguna, Santa Cruz de Tenerife, Spain; alu0101534848@ull.edu.es (M.B.Y.); jchaopel@ull.edu.es (J.C.-P.); alu0101534855@ull.edu.es (A.O.); isifaoui@ull.edu.es (I.S.); jpinero@ull.edu.es (J.E.P.); 2 Laboratory of Functional Physiology and Valorization of Bio-Resources, Higher Institute of Biotechnology of Beja, University of Jendouba, Beja 382-9000, Tunisia; sebaihichem@yahoo.fr; 3 Instituto Universitario de Bio-Orgánica Antonio González, Departamento de Química Orgánica, Universidad de La Laguna, Avenida Astrofísico Francisco Sánchez 2, 38206 La Laguna, Tenerife, Spain; alu0100947311@ull.edu.es (E.H.-Á.); ilopez@ull.edu.es (I.L.B.); 4 Departamento de Obstetricia y Ginecología, Pediatría, Medicina Preventiva y Salud Pública, Toxicología, Medicina Legal y Forense y Parasitología, Universidad de La Laguna, C/Sta. María Soledad s/n, 38200 San Cristóbal de La Laguna, Santa Cruz de Tenerife, Spain; 5 Consorcio Centro de Investigación Biomédica en Red, Área de Enfermedades Infecciosas (CIBERINFEC), Instituto de Salud Carlos III, Av. Monforte de Lemos 3-5, Pabellón 11, 28029 Madrid, Spain

**Keywords:** *Mentha rotundifolia*, ursolic acid, Primary Amoebic Meningoencephalitis, structure-activity relationship, mechanism of action

## Abstract

Current therapies for Primary Amoebic Meningoencephalitis (PAM) present several limitations; consequently, there is an urgent need to develop new therapeutic agents. In this regard, we undertook bioassay-guided isolation of *Mentha rotundifolia* leaves which allowed the identification of ursolic acid (**1**) as the main bioactive metabolite against two ATCC strains of *Naegleria fowleri* (ATCC^®^ 30808^TM^ and ATCC^®^ 30215^TM^). Moreover, ten ursolic acid derivatives (**2**–**11**) were synthesized through esterification and nucleophilic substitution to obtain halo or amino ester derivatives. Among these derivatives, compound **7** exhibited the highest activity against the *N. fowleri* ATCC^®^ 30808^TM^ strain with an IC_50_ value of 28.66 µM, whereas compound **10** showed the top activity against the *N. fowleri* ATCC^®^ 30215^TM^ strain with an IC_50_ of 7.61 µM, surpassing the efficacy of the reference drug, miltefosine. Both compounds, **7** and **10**, showed a good selectivity index and hence low toxicity in vitro. Structure–activity relationship studies revealed that the type of acylating agent played a crucial role in the activity. Furthermore, both compounds induced autophagy and apoptosis-like processes in both treated *N. fowleri* strains. These results highlight the potential of ursolic acid-related triterpenoids as drug scaffolds and identify *M. rotundifolia* as a promising natural source of amoebicidal agents against PAM.

## 1. Introduction

Free-living amoebae of the genus *Naegleria* are widespread in soil and freshwater environments, including rivers, lagoons, hot springs, and poorly treated pools. *Naegleria fowleri*, known as the “brain-eating amoeba,” is a thermophilic unicellular organism in the phylum Percolozoa that can survive temperatures up to 45 °C, enabling it to thrive in warm freshwater of tropical and subtropical regions. Like other *Naegleria* species, it has two life stages: the trophozoites and the cyst, and some species form a transient flagellated stage to move rapidly under unfavorable conditions. Of the 47 recognized species, *N. fowleri* is the only one causing Primary Amoebic Meningoencephalitis (PAM), a rapidly progressing and almost always fatal CNS infection [1,2]. PAM arises when trophozoites enter the nasal cavity and reach the brain via olfactory nerves, causing severe inflammation and intracranial pressure. Although rare, with roughly 400 cases reported worldwide, fatality exceeds 95%. Most cases occur in the United States, Pakistan, and India, typically linked to swimming in contaminated water or nasal exposure during ablution. Environmental and human factors, such as poor water maintenance, climate change, and industrialization, facilitate its spread [3,4].

Early PAM symptoms resemble viral or bacterial meningitis, headache, fever, nausea, stiff neck, progressing rapidly to confusion, seizures, coma, and death. Because they are nonspecific, diagnosis is often delayed, lowering survival chances. Treatment options are very limited: even with early use of amphotericin B or miltefosine, outcomes are rarely successful due to low efficacy and severe side effects. Despite increasing research efforts, major knowledge gaps in PAM biology, pathogenesis, and control remain. These gaps are due to the disease’s rarity, the rapid progression and high fatality that limit the availability of clinical samples, the difficulty of maintaining reliable in vitro and in vivo infection models, and the limited funding typically available for rare diseases [3,4,5,6]. Despite ongoing research [5], no vaccine is currently available, and chemotherapy remains the primary treatment strategy [6]. Numerous synthetic and natural compounds are under investigation for activity against *N. fowleri*. Nevertheless, a comprehensive review that synthesizes recent advances and identifies critical gaps in our understanding of its biology, pathogenesis, and control is still lacking [6,7,8,9,10].

Nature has long inspired major advances in healthcare [11], with natural compounds remaining a key source of modern drugs due to their structural diversity and biological specificity [12]. Despite advances in synthetic chemistry and high-throughput screening, natural products are especially important in therapies for cancer and bacterial and parasitic diseases. From 1981 to 2019, over half of approved anticancer, anti-infective, and antidiabetic drugs were natural products or derivatives, highlighting their central role in drug discovery [12]. Their unique chemical scaffolds, often difficult to reproduce synthetically, make them particularly valuable for developing novel therapeutic agents [13,14].

*Mentha*, a genus of the Lamiaceae family comprising about 20 species and several hybrids, has been globally distributed and valued since antiquity for its medicinal, aromatic, and culinary uses [15]. *Mentha* species are rich in terpenes and other metabolites, such as cinnamic acids, flavonoids, and polysaccharides, making them valuable in traditional medicine and cosmetic and culinary applications [16]. Recent studies have also explored the anti-kinetoplastid potential of *Mentha* species [17,18]. In Tunisia, this genus is represented by several species, including *Mentha rotundifolia* (L.) Huds [19,20], which has been used in Tunisian traditional medicine for its therapeutic properties, especially its essential oil, as an antimicrobial, insecticide, neuroprotective, and antioxidant [21,22,23]. The impact of *M. rotundifolia* on *Naegleria* remains unexplored, with only three patents linking the genera, highlighting a neglected avenue in parasitology.

Continuing our research on discovering novel amoebicidal agents, we report herein the isolation of ursolic acid (**1**) through a bioassay-guided fractionation of *M. rotundifolia* leaf extract against *N. fowleri*. Furthermore, ten ursolic acid derivatives (**2**–**11**) were synthesized, and structure–activity relationship (SAR) studies were conducted to identify the key functional groups responsible for their amoebicidal activity. Additionally, the mechanism of action of the most promising compounds (**7** and **10**) was investigated to better understand their molecular interactions and support their therapeutic potential.

## 2. Results

### 2.1. Bioassay-Guided Isolation

**Figure 1:**
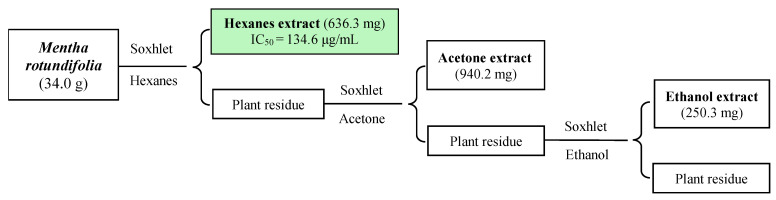
Flowchart of the *M. rotundifolia* leaves extraction using polarity gradient solvents. Antiparasitic activity was evaluated against *N. fowleri* ATCC^®^ 30808^TM^ trophozoites. IC_50_: Concentration able to inhibit 50% of the growth of the tested parasite, expressed in µg/mL.

The objective of the present study was to conduct bioprospecting of *M. rotundifolia* as a potential source of natural amoebicidal agents through a bioactivity-guided fractionation. Firstly, a small amount of *M. rotundifolia* leaves (34.0 g) was sequentially extracted using organic solvents of increasing polarity, hexanes (Hx), acetone, and ethanol (EtOH). The obtained extracts were evaluated for their amoebicidal activity against *N. fowleri* ATCC^®^ 30808^TM^ trophozoites. For comparative purposes, miltefosine (IC_50_ = 12.73 μg/mL) and amphotericin B (IC_50_ = 0.18 μg/mL) were tested against the same strain (Figure 1, Table 1).

**Figure 2:**
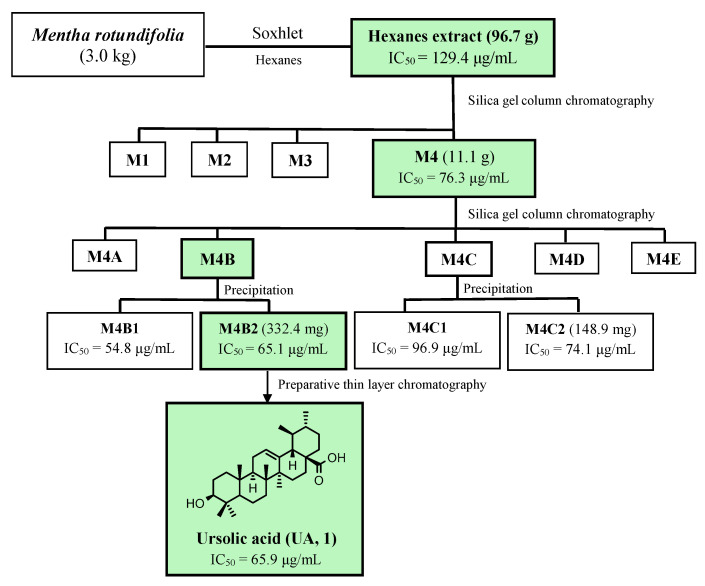
Flowchart of antiprotozoal bio-guided fractionation of hexanes extract of *M. rotundifolia* against *N. fowleri* ATCC^®^ 30808™ trophozoites. IC_50_: Concentration able to inhibit 50% of the growth of the tested parasite, expressed in µg/mL.

The results showed that the hexanic extract exhibited the most potent amoebicidal profile, with an IC_50_ value of 134.59 μg/mL on *N. fowleri* ATCC^®^ 30808™, suggesting an enrichment in bioactive compounds. Based on these promising preliminary results, a comprehensive phytochemical investigation was carried out through a bioassay-guided fractionation in a scaled-up study. For this purpose, 3.0 kg of leaves was selectively extracted using hexanes. The resulting extract was then subjected to further fractionation by silica gel column chromatography (CC) and thin-layer chromatographic (TLC) analysis, affording four fractions (M1–M4), which were screened for their biological activity (Figure 2, Table 1).

Fraction M4 showed the most promising anti-amoebic activity with an IC_50_ value of 76.28 μg/mL on *N. fowleri* ATCC^®^ 30808™. This fraction was then fractioned by silica gel CC, affording five sub-fractions (M4A-M4E). Sub-fractions M4B and M4C were fractionated by precipitation, affording the sub-fractions M4B1, M4B2, M4C1, and M4C2 (Figure 2). The amoebicidal activity of all the sub-fractions revealed that the most promising were sub-fractions M4B1, M4B2, M4C1, and M4C2 with IC_50_ values ranging from 96.91 to 54.75 μg/mL. Among the four sub-fractions, M4B2 was selected for further study based on its biological activity and phytochemical profile, as revealed by thin-layer chromatography (TLC). This subfraction was subsequently purified by preparative TLC to yield ursolic acid (UA, **1**), which was identified by its spectroscopic data and comparison with previously reported data [24]. UA was evaluated, exhibiting promising amoebicidal effects with an IC_50_ value of 65.91 μg/mL against *N. fowleri* ATCC^®^ 30808™, which encouraged us to conduct further studies.

To our knowledge, ursolic acid is an amoebicidal agent, underscoring its potential as a promising therapeutic compound. This naturally occurring triterpene has demonstrated significant efficacy against *N. fowleri* and *Balamuthia mandrillaris* free-living amoeba, opportunistic protists responsible for PAM and granulomatous amoebic encephalitis, respectively. The observed effectiveness of ursolic acid reinforces the value of plant-derived compounds as potential therapeutic agents against infections caused by free-living amoebae, positioning this natural compound as a strong candidate for the development of novel amoebicidal agents [25].

### 2.2. Chemical Modification

**Figure 3:**
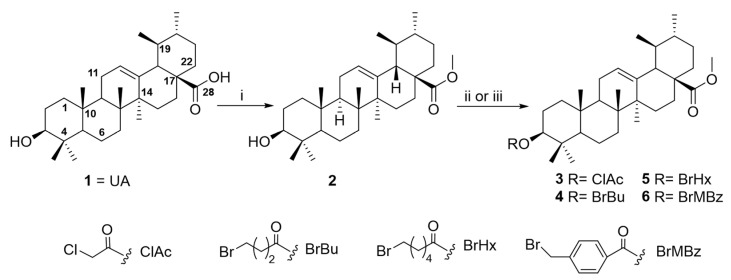
Preparation of halo-ester ursolic acid derivatives (**2**–**6**). Reagents and conditions. i: CH_3_I, K_2_CO_3_, DMF, N_2_, rt. ii: Chloroacetic anhydride or 4-bromobutanoyl chloride or 6-bromohexanoyl chloride, Et_3_N, DMAP, CH_2_Cl_2_, N_2_, rt. iii: 4-bromomethylbenzoic acid, DMAP, DCC, N_2_, rt.

**Figure 4:**
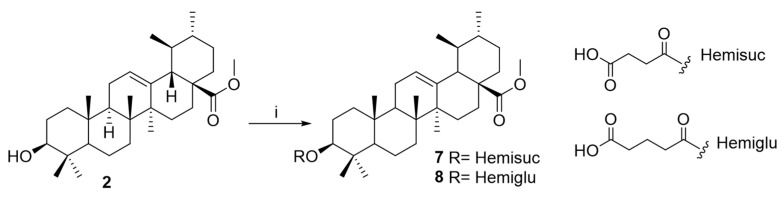
Preparation of carboxyl-ester ursolic acid derivatives (**7**–**8**). Reagents and conditions. i: succinic or glutaric anhydride, Et_3_N, DMAP, DMF, N_2_, rt.

**Figure 5:**
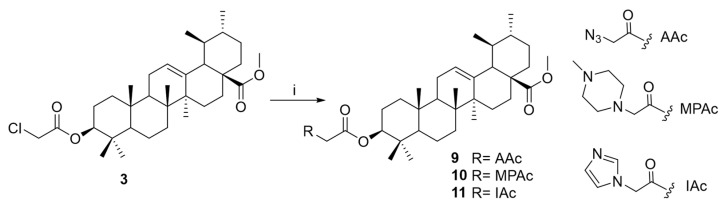
Preparation of azido-ester or amino-ester ursolic acid derivatives (**9**–**11**). Reagents and conditions. i: NaN_3_, or imidazole or N-methylpyperazine, KF, CH_3_CN, N_2_, rt.

A comprehensive approach was conducted to optimize ursolic acid as a lead compound, aiming to gain insights into the structure–activity relationships (SARs) of this pentacyclic triterpenoid as a potential amoebicidal agent. To enable rational structural modification, the UA molecule has been analyzed in terms of its functional groups and pharmacophores, which are critical for its biological activity. These sites are broadly categorized as the carboxylic moiety (C-28), hydroxy function (C-3), and an alkene at C-12/C-13. The present study was focused on UA derivatives modified at C-3 and C-28 positions. Firstly, methyl ursolate (**2**) was synthesized by the reaction of UA (**1**) with methyl iodide. Subsequently, the methyl ursolate (**2**) underwent esterification with a series of carboxylic acid derivatives, yielding the corresponding halo-ester derivatives (**3**–**6**) and carboxyl-ester (**7**–**8**) (Figure 3 and Figure 4). Finally, 3-*O*-chloroacetyl-methyl ursolate (**3**) was modified to yield the azido-ester (**9**) and amino-ester (**10**–**11**) derivatives (Figure 5). Notably, derivatives **5**–**11** are reported for the first time, representing novel contributions to the field. The structure of the known compound methyl ursolate (**2**) [26] was identified by spectroscopic methods and comparison with reported data. Moreover, although compounds **3** [27] and **4** [28] have been previously described, their spectroscopic data have not yet been reported. Therefore, this study provides, for the first time, their detailed spectroscopic data (Supporting Information, pages S2–S12).

### 2.3. In Vitro Amoebicidal Activity and Cytotoxicity of Ursolic Acid Derivatives

UA (**1**) and its derivatives **2**-**11** were evaluated for their in vitro amoebicidal activity against ATCC strains of *Naegleria fowleri* (ATCC^®^ 30808^TM^ and ATCC^®^ 30215^TM^). Furthermore, their cytotoxicity were assessed using the murine macrophage cell line J774.A1 to determine their safety profiles. For comparative analysis, the reference drugs miltefosine and amphotericin B were also included in the evaluation.

Amoebicidal activity (Table 2) revealed that five compounds exhibited moderate to potent activity against *N. fowleri* ATCC^®^ 30808™, with IC_50_ values ranging from 28.66 to 88.70 μM. All five compounds were more active than ursolic acid (UA, **1**), which showed an IC_50_ value of 141.53 μM. Notably, compound **7** displayed the highest potency, with an IC_50_ of 28.66 μM, outperforming the reference drug miltefosine (IC_50_ = 38.74 μM). In addition to its potency, compound **7** exhibited an acceptable selectivity profile, with a selectivity index (SI) of 6.1. Moreover, five compounds demonstrated moderate to potent amoebicidal activity against *N. fowleri* ATCC^®^ 30215™, with IC_50_ values ranging from 7.61 to 84.47 μM. Among them, four compounds exhibited greater potency than the ursolic acid (UA, IC_50_ = 82.53 μM) and the miltefosine (IC_50_ = 81.57 μM). In contrast, the remaining compounds showed limited or no activity, with IC_50_ values exceeding 100 μM. Compound **10** emerged as the most active, with an IC_50_ of 7.61 μM, representing more than a 10-fold increase in potency compared to miltefosine and UA. Additionally, compound **10** exhibited good selectivity, with a selectivity index of 7.6.

### 2.4. Structure–Amoebicidal Activity Relationship Study

**Figure 6:**
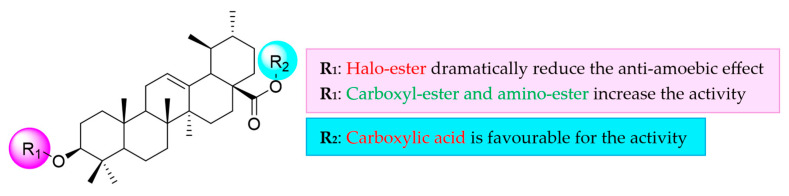
Preliminary structure–activity relationship analysis of ursolic acid derivatives.

A preliminary structure–activity relationship analysis showed that the ester moiety is essential for the amoebicidal activity (Figure 6). The results of UA (**1**) and methyl ursolate (**2**) indicate that methylation of the carboxylic acid leads to an increase in activity against *N. fowleri* ATCC^®^ 30808™ trophozoites, while the activity remains very similar in *N. fowleri* ATCC^®^ 30215™. Furthermore, the halo-esters (**3**-**6**) synthesized by esterification of the secondary alcohol at the C-3 position of methyl ursolate (**2**) exhibited drastically reduced activity; they were inactive compounds (IC_50_ > 200 μM). The amoebicidal activity results of the carboxy ester derivatives (**7**-**8**) clearly demonstrate that the conversion of methyl ursolate (**2**) into its hemisuccinate or hemiglutarate derivatives leads to an enhanced activity. Notably, increasing the number of carbon atoms in the side chain from three to four (i.e., from compound **7** to **8**) further improves the amoebicidal effect in *N. fowleri* ATCC^®^ 30808™, while the activity remains similar in *N. fowleri* ATCC^®^ 30215™. Furthermore, the nucleophilic substitution of chlorine with azide from the chloroacetate methyl ursolate (**3**) does not produce any change in activity, as this derivative (**9**) was inactive. However, derivatives prepared using *N*-methyl piperazine (**10**) or imidazole (**11**) resulted in a drastic increase in activity. The present structural modification resulted in two of the most active derivatives across both strains. Overall, the SAR data highlight compounds **7** and **10** as potent and selective agents, making them attractive candidates for further optimization.

### 2.5. In Vitro Cysticidal Activity on Naegleria fowleri

Based on the IC_50_ values of UA (**1**) and its derivatives (**2**–**11**) on *N. fowleri* trophozoites, derivatives **7** and **10** were selected for cysticidal activity against both *N. fowleri* strains. Furthermore, the murine macrophage cell line J774.A1, miltefosine, and amphotericin B were also evaluated for safety and comparative purpose (Table 3).

Against the ATCC^®^ 30808™ strain, both compounds showed lower activity than the positive controls miltefosine and amphotericin B, with IC_50_ values of 36.65 ± 1.49 µM and 66.47 ± 20.64 µM, respectively. Conversely, against the ATCC^®^ 30215™ strain, both compounds were more active than miltefosine but less active than amphotericin B, showing IC_50_ values of 28.03 ± 1.59 µM (compound **7**) and 17.14 ± 2.59 µM (compound **10**). Notwithstanding, both compounds exhibited an acceptable selectivity profile, except for compound **10** against the ATCC^®^ 30808™ strain.

### 2.6. Investigation into the Molecular Mechanisms of Compounds *7* and *10*

Based on the results of in vitro amoebicidal activity assays against both trophozoite strains and the cyst-resistant stage of *N. fowleri*, as well as cytotoxicity evaluation in murine J774A.1 macrophages, compounds **7** and **10** were selected for further investigation of their mechanisms of action. For all subsequent assays, *N. fowleri* ATCC^®^ 30808™ and ATCC^®^ 30215™ trophozoites were treated with IC_90_ concentrations of compounds **7** (39.53 µM) and **10** (17.32 µM).

**Figure 7:**
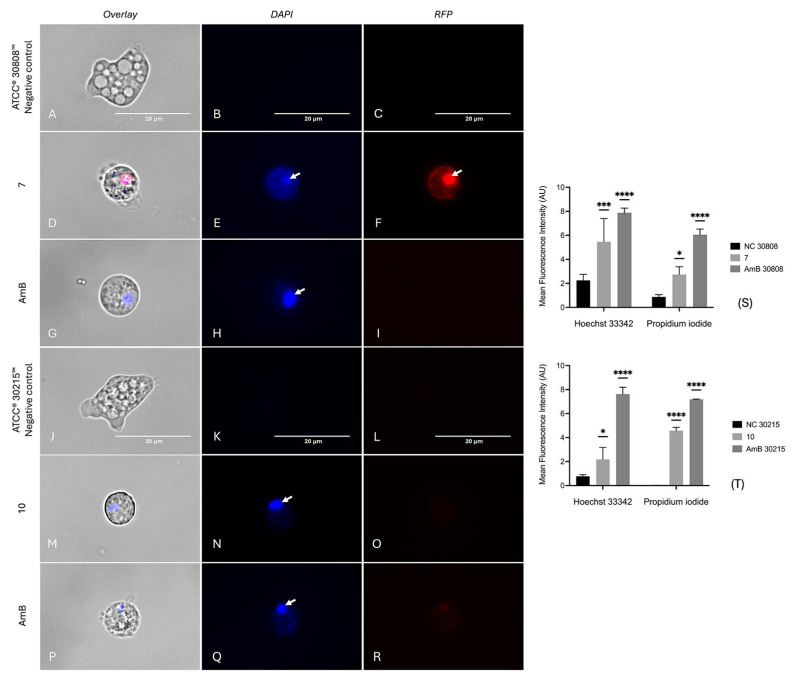
Detection of chromatin condensation in N. fowleri trophozoites using Hoechst and propidium iodide staining. Chromatin condensation was assessed in *N. fowleri* ATCC^®^ 30808™ treated with compound **7** (panels **D**–**F**) and *N. fowleri* ATCC^®^ 30215™ treated with compound **10** (panels **M**–**O**), compared with the positive control amphotericin B (panels **G**–**I** and **P**–**R**, respectively) and untreated negative controls (panels **A**–**C** and **J**–**L**). Arrows highlight areas with bright blue Hoechst fluorescence, corresponding to chromatin condensation, and red PI fluorescence, indicating loss of plasma membrane integrity. Cells were stained with Hoechst and propidium iodide, and images were acquired at 100× magnification using an EVOS™ M5000 microscope with DAPI, RFP, and overlay channels. Scale bar: 20 µm. Fluorescence intensity (arbitrary units) was quantified from three independent fields and analyzed using one-way ANOVA (**S**,**T**). Data are presented as mean ± SD (*n* = 3); * *p* < 0.05; *** *p* < 0.001; **** *p* < 0.0001, indicating statistically significant differences versus the negative control.

The condensation of the chromatin is one of the most typical hallmarks of apoptotic cell death [29]. In both strains, the negative controls (Figure 7A–C and Figure 7J–L, respectively) exhibited only faint Hoechst 33,342 (blue) and PI (red) fluorescence, consistent with healthy nuclear morphology and intact membranes. In contrast, treatments with compounds **7** (Figure 7D–F), **10** (Figure 7M–O), and amphotericin B (positive control, Figure 7G–I,P–R) showed a clear chromatin condensation, as revealed by intense Hoechst 33,342 fluorescence observed in all trophozoites. In cells treated with compound **7**, in addition to blue color emission, strong red fluorescence is observed (Figure 7E–F), consistent with a late apoptosis stage. Quantification of Hoechst and PI fluorescence (Figure 7S–T) revealed significant increases in both nuclear condensation and membrane damage in *N. fowleri* treated with both compounds.

**Figure 8:**
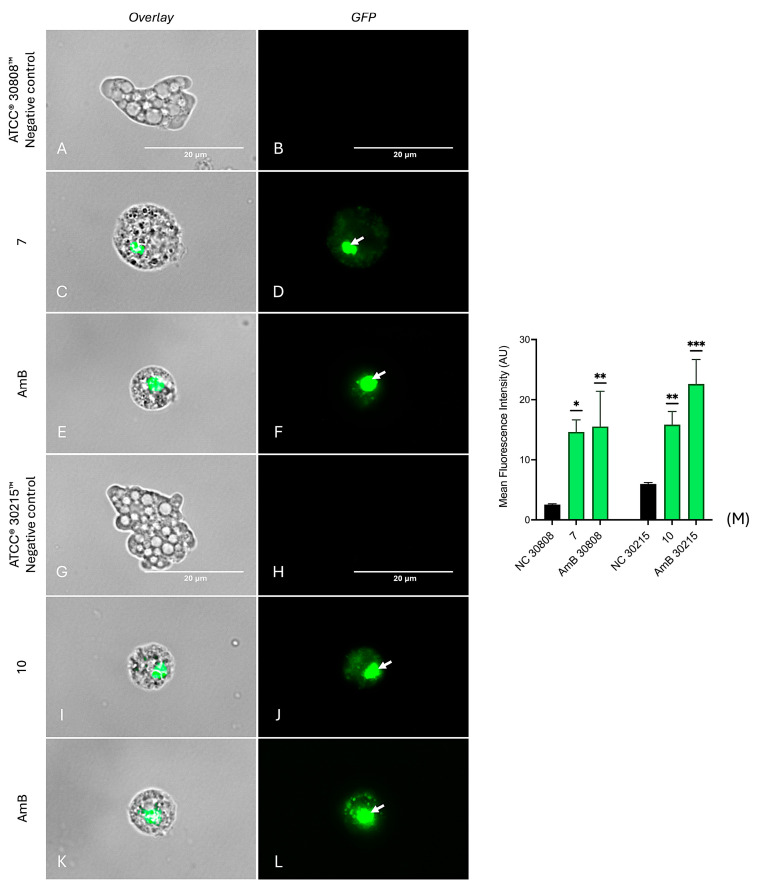
Assessment of plasma membrane integrity in *N. fowleri* trophozoites using SYTOX™ Green staining. Plasma membrane permeabilization was evaluated following treatment with the IC90 of compound **7** in *N. fowleri* ATCC^®^ 30808™ (panels **C**,**D**), compound **10** in *N. fowleri* ATCC^®^ 30215™ (panels **I**,**J**), and amphotericin B as a positive control (panels **E**,**F**,**K**,**L**). Arrows highlight areas with strong green fluorescence, corresponding to cells that lost plasma membrane integrity. In contrast, negative controls (panels **A**,**B**,**G**,**H**) showed no fluorescence, confirming intact membranes. Images were acquired at 100× magnification using the EVOS™ M5000 microscope with GFP and overlay channels. Scale bar: 20 µm. Quantification of SYTOX™ Green-positive cells is presented in panel (**M**). Data represent mean ± SD (*n* = 3), analyzed by one-way ANOVA; * *p* < 0.05; ** *p* < 0.01; *** *p* < 0.001 versus negative control.

Plasma membrane integrity of *N. fowleri* trophozoites was analyzed using SYTOX^®^ Green, a membrane-impermeable dye that fluoresces upon binding to nucleic acids in cells with compromised membranes [10,30]. Negative controls from both strains emit low fluorescence (Figure 8A,B,G,H), indicating intact membranes. In contrast, trophozoites treated with compounds **7** (Figure 8C,D) and **10** (Figure 8I,J) resulted in strong green fluorescence, indicating significant membrane disruption. The positive control, amphotericin B, also induced bright green fluorescence in both strains (Figure 8E,F,K,L), consistent with its known membrane permeabilizing activity. Quantitative analysis of fluorescence intensity (Figure 8M) showed that our compounds induced a statistically significant increase in fluorescence compared to the negative control, confirming their membrane disruptive effects. However, as can be seen under transmitted light, the integrity and cellular shape of the trophozoites was maintained, ruling out a process of cell death due to necrosis. Although SYTOX Green and propidium iodide (PI) share the same cellular target, the nucleic acids, SYTOX Green has demonstrated higher binding efficiency in cells with compromised membranes, likely due to its enhanced ability to penetrate through pores formed by increased membrane permeability.

**Figure 9:**
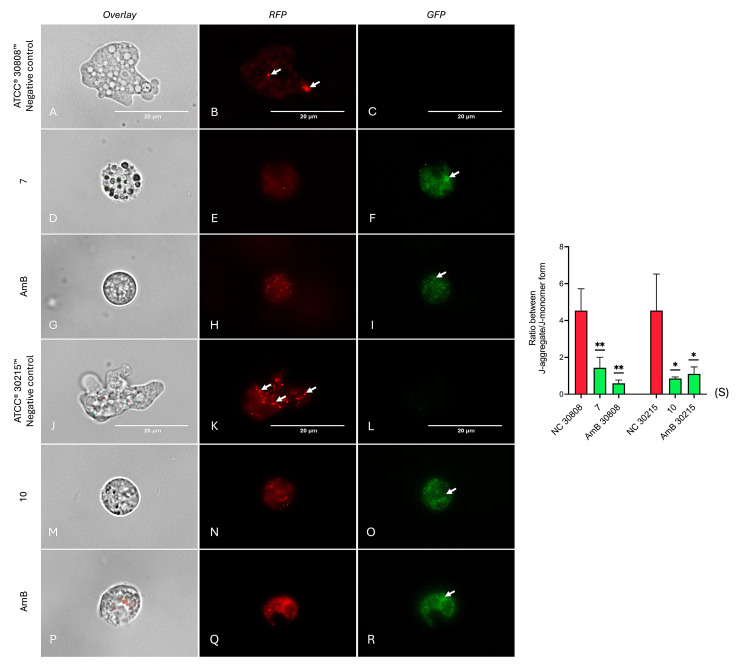
Evaluation of mitochondrial membrane potential in *N. fowleri* trophozoites using JC-1 dye. Representative fluorescence microscopy images show the effects of compound **7** on *N. fowleri* ATCC^®^ 30808™ (panels **D**–**F**) and compound 10 on *N. fowleri* ATCC^®^ 30215™ (panels **J**–**L**), compared with the positive control amphotericin B (**G**–**I** and **P**–**R**, respectively) and untreated negative controls (**A**–**C** and **M**–**O**). Arrows indicate areas where JC-1 fluorescence shifts from red to green, reflecting a decrease in mitochondrial membrane potential. Cells were stained with JC-1, and images were captured at 100× magnification using an EVOS™ M5000 microscope with RFP, GFP, and merged channels. Scale bar: 20 µm. Fluorescence intensity was quantified from three independent fields and analyzed by one-way ANOVA (**S**). Data are presented as mean ± SD (*n* = 3); * *p* < 0.05; ** *p* < 0.01; versus negative control.

The mitochondrial membrane potential of amoebae was evaluated using a JC-1 mitochondrial membrane potential assay kit. The results (Figure 9) revealed that compounds **7** and **10** induced a marked decrease in mitochondrial membrane potential, similar to the effect observed with the positive control, amphotericin B (Figure 9G–I,P–R). While the negative control exhibited intense red fluorescence (Figure 9B,K), indicative of healthy mitochondrial function, trophozoites treated with compound **7** (Figure 9D–F) and compound **10** (Figure 9M–O) predominantly showed green fluorescence. This fluorescence shift is consistent with JC-1 accumulation in its monomeric form due to mitochondrial depolarization, reflecting a loss of mitochondrial membrane potential and suggesting the induction of mitochondrial dysfunction, likely contributing to apoptosis [10]. Furthermore, quantitative analysis (Figure 9S) showed that our compounds significantly depolarized the mitochondrial membrane in *N. fowleri*, as indicated by increased green fluorescence, comparable to amphotericin B. These results were statistically significant (*p* < 0.01 or *p* < 0.001).

**Figure 10:**
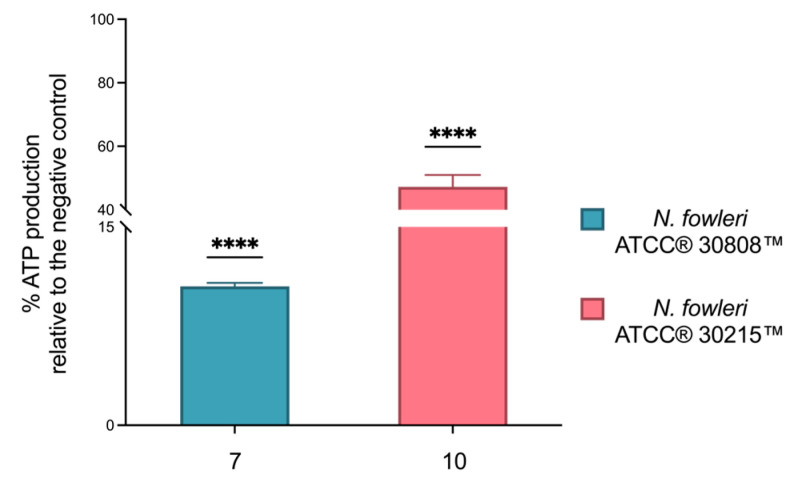
Relative ATP production measured in *N. fowleri* trophozoites by CellTiter-Glo^®^ assay. ATP levels were quantified following treatment with compounds **7** and **10** and compared to the negative control, which represents 100% ATP production in healthy, untreated cells. Data are presented as mean ± SD (*n* = 3). Statistical significance was determined using one-way ANOVA; **** *p* < 0.0001 versus negative control.

In addition, the impact on mitochondrial function was further supported by the analysis of intracellular ATP levels. As shown in Figure 10, the results are expressed as the percentage inhibition of ATP levels compared to the negative control (set as 100% ATP). Compounds **7** and **10** caused a significant reduction in ATP production, with an inhibition of 89.5% and 47.3%, respectively.

**Figure 11:**
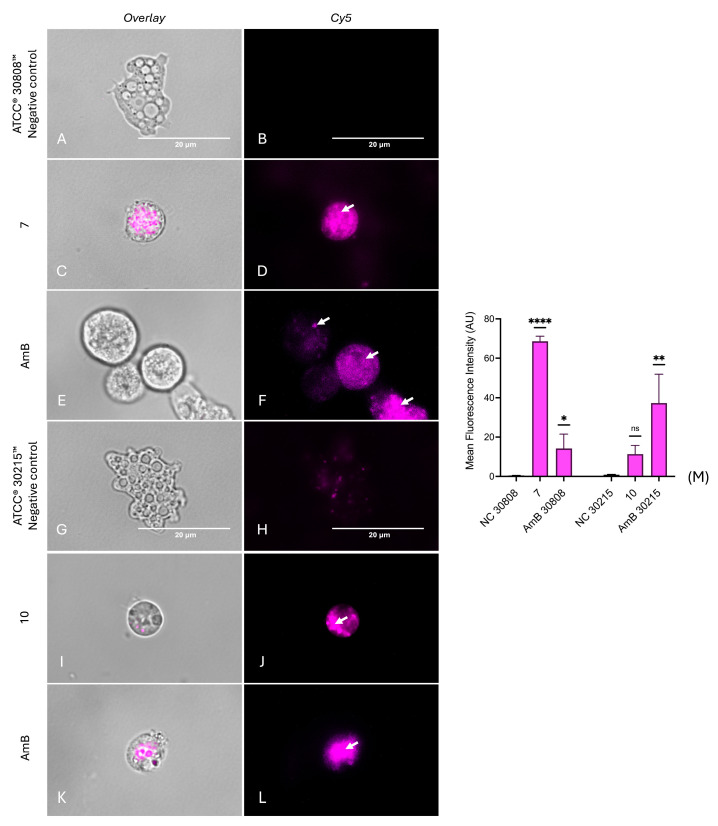
Detection of reactive oxygen species accumulation in *N. fowleri* trophozoites using CellROX™ Deep Red staining. ROS generation was assessed in *N. fowleri* ATCC^®^ 30808™ treated with compound **7** (panels **C**,**D**) and *N. fowleri* ATCC^®^ 30215™ treated with compound **10** (panels **G**,**H**), compared to amphotericin B as a positive control (panels **E**,**F**,**K**,**L**) and untreated negative controls (panels **A**,**B**,**I**,**J**). Arrows highlight regions with more intense red fluorescence, corresponding to regions of elevated ROS accumulation. Cells were stained with CellROX™ Deep Red and imaged at 100× magnification using the EVOS™ M5000 microscope with Cy5 and overlay channels. Scale bar: 20 µm. Fluorescence intensity (arbitrary units) was quantified from three independent fields and analyzed using one-way ANOVA (**M**). Data are presented as mean ± SD (*n* = 3); ns: non-significant; * *p* < 0.05; ** *p* < 0.01; **** *p* < 0.0001 versus negative control.

Reactive oxygen species (ROS) are key indicators of cellular stress, as their accumulation disrupts cellular homeostasis and triggers cell death pathways [31]. As indicated in Figure 11, both *N. fowleri* strains displayed intense magenta fluorescence following treatment, corresponding to an increase in the intracellular ROS accumulation after 24 h, compared with the positive (Figure 11E,F,K,L) and negative controls (Figure 11A,B,G,H) of both strains. Moreover, fluorescence quantification (Figure 11) showed that compound **7** significantly increased ROS in strain ATCC^®^ 30808™ (*p* < 0.05), while compound **10** had a significant effect on strain ATCC^®^ 30215™ (*p* < 0.01), confirming oxidative stress induction by both compounds. Thus, a clear accumulation of ROS inside trophozoites was evidenced, confirming that both compounds trigger oxidative stress in this amoeba.

**Figure 12:**
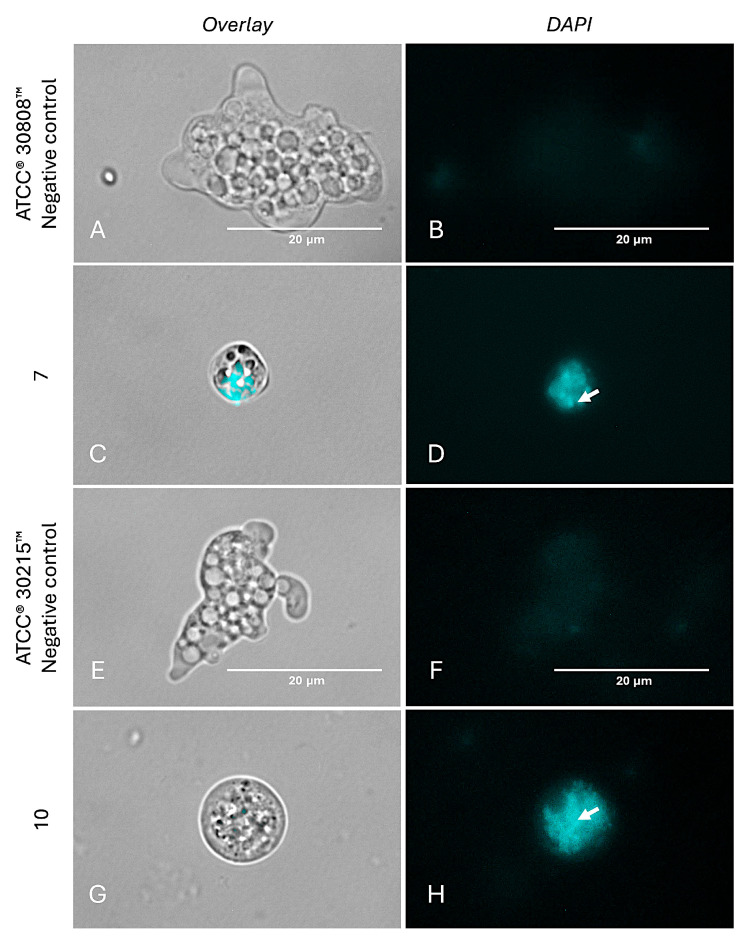
Visualization of autophagic vesicles in *N. fowleri* trophozoites. Autophagic activity was assessed by staining autophagic compartments with monodansylcadaverine (MDC), which selectively labels autophagic vesicles in treated cells (**C**,**D**,**G**,**H**). Untreated cells exhibit low blue fluorescence (**A**,**B**,**E**,**F**). Arrows indicate autophagosomes where the reagent accumulates, resulting in a more intense cyan-blue fluorescence. Images were captured using the EVOS™ M5000 microscope at 100× magnification with DAPI and overlay channels. Scale bar: 20 μm.

Moreover, autophagy represents an additional programmed cell death (PCD) pathway that has recently been reported in several parasites, including *N. fowleri* [8]. Treatment with compounds **7** (Figure 12C,D) and **10** (Figure 12G,H) led to the appearance of distinct cyan-blue fluorescence throughout the cytoplasm, indicative of autophagosome formation containing degraded cellular components and organelles. In contrast, the negative control (Figure 12A,B,E,F) exhibited only a diffuse background signal from the staining reagent, consistent with the absence of autophagic activity.

**Figure 13:**
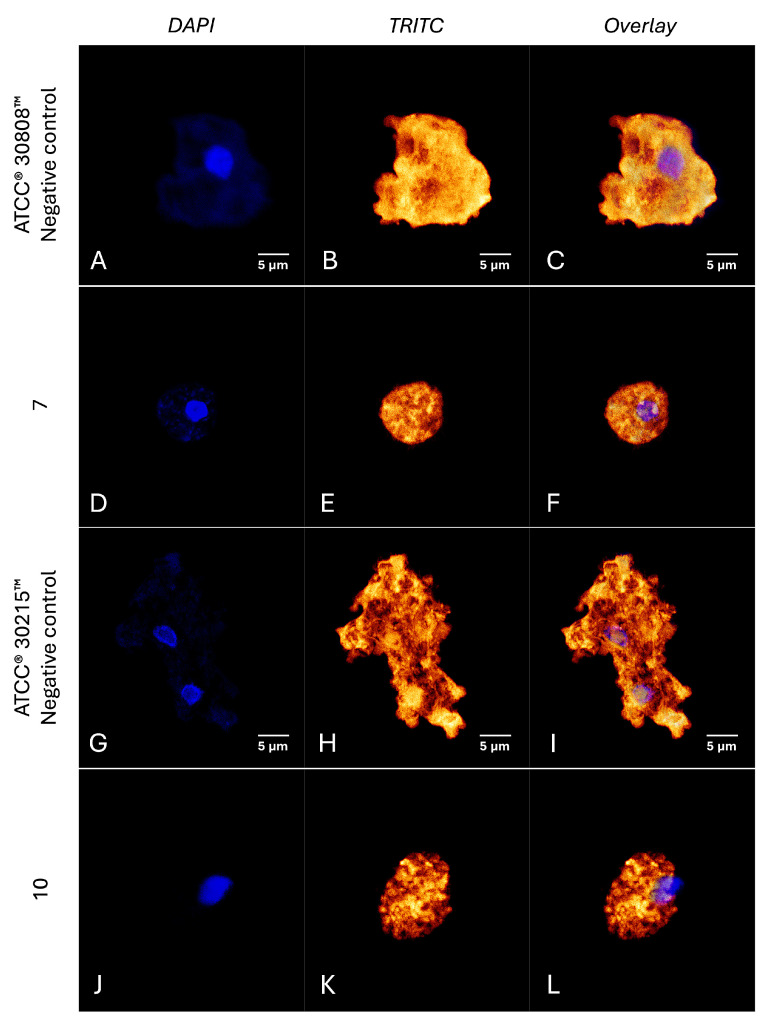
Disruption of the actin cytoskeleton in *N. fowleri* trophozoites. Actin filaments were visualized using phalloidin-TRITC stained in orange/red (**B**,**E**,**H**,**K**), and nuclei were counterstained with DAPI emitting blue fluorescence (**A**,**D**,**G**,**J**) in *N. fowleri* ATCC^®^ 30808™ treated with compound **7** and *N. fowleri* ATCC^®^ 30215™ treated with compound **10**, Merge DAPI and TRITC Channels (**C**,**F**,**I**,**L**). Treatment with the IC_90_ of compounds **7** and **10** resulted in reduced actin signal intensity and morphological alterations compared to untreated controls. Images were acquired using a Leica DMI4000 B confocal microscope (Leica Microsystems, Wetzlar, Germany) with LAS X software 3.5.5.19976, employing 405 nm and 532 nm lasers and a Leica HCX PL Apo 63× oil immersion objective. Scale bar: 5 μm.

Confocal fluorescence microscopy revealed that trophozoites treated with the compounds exhibited a pronounced disruption of actin organization, evidenced by a significant reduction in fluorescence intensity compared to the negative controls (Figure 13). This alteration suggests extensive actin depolymerization or cytoskeletal disorganization, potentially impairing essential cellular functions such as motility and structural integrity [32].

## 3. Discussion

Medicinal plants remain a promising source of bioactive compounds for the discovery and development of new antiparasitic agents [33]. *Mentha rotundifolia*, a species well-documented for its diverse phytochemical composition and broad spectrum of biological activities [34], was found to contain ursolic acid, a bioactive triterpenoid previously shown to induce regulated cell death mechanisms [35]. In an effort to increase efficacy, structurally modified derivatives of ursolic acid (UA) were designed, synthesized, and evaluated against the *N. fowleri*, the causative agent of Primary Amoebic Meningoencephalitis, for which effective treatments remain scarce. Among the tested derivatives, compounds **7** and **10** exhibited potent activity against the infectious stage of the pathogen. Notably, they also showed effectiveness against cysts, which are typically resistant to treatment due to their thick carbohydrate-rich wall [36]. This dual-stage efficacy suggests that the compounds may either penetrate the cyst wall through existing pores or disrupt essential structural components, such as wall-associated enzymes or proteins [32]. Moreover, multiple metabolic events related to different PCD pathways in strains ATCC^®^ 30808™ and ATCC^®^ 30215™ were demonstrated. Apoptosis-like features were evidenced by decreased ATP levels, mitochondrial membrane depolarization, chromatin condensation, increased membrane permeability, and enhanced oxidative stress due to elevated ROS levels, all hallmarks that are indicators of PCD previously reported in protozoa.

The cytoskeleton plays a crucial role in maintaining cell shape, motility, intracellular transport, and overall structural integrity. In pathogenic protozoa, it is also essential for adhesion, migration, and host invasion. Given its central role in parasite viability and infectivity, the cytoskeleton represents a promising therapeutic target for antiparasitic drug development. In this context, phalloidin-TRITC staining revealed significant disruption of the actin cytoskeleton following treatment, including filament disassembly and actin cluster formation, suggesting that actin structures may represent a direct molecular target of these compounds. Furthermore, treated cells exhibited marked morphological alterations, such as cell shrinkage and reduced volume, indicative of compromised cytoskeletal integrity [32]. These disruptions are likely to impair critical cellular functions, including adhesion and motility, thereby contributing to the amoebicidal effects observed. In addition to apoptosis, autophagy has emerged as a complementary form of PCD in protozoan parasites, particularly under conditions of chemical or environmental stress. This process allows the degradation of damaged organelles and cytoplasmic components through the formation of autophagosomes, contributing to parasite elimination when excessively activated. In our study, the presence of autophagic vacuoles, evidenced by distinct cyan-blue fluorescence in treated cells, further supports the induction of autophagy as a complementary cell death mechanism. The simultaneous induction of both apoptotic and autophagic markers suggests that *N. fowleri* may activate overlapping PCD pathways in response to triterpenoid treatment. These findings are in line with previous reports demonstrating that related compounds can trigger multiple cell death mechanisms in *Naegleria* species [8,32]. The convergence of our results with prior research reinforces the therapeutic potential of these UA derivatives against free-living amoebae.

Observed apoptotic and autophagy-like responses in *N. fowleri* treated with UA derivatives corroborate accumulating data on the multifaceted bioactivity of UA and related triterpenoids across a range of biological systems. Ursolic acid has been extensively characterized as a bioactive scaffold with notable antineoplastic activity, with the ability to induce programmed cell death in various human cancer cell types and in vivo models [37,38]. In particular, UA has been shown to inhibit cell proliferation and promote apoptosis in oral, colorectal, and lung cancer cells. Beyond oncology, similar apoptosis-like mechanisms have been reported in protozoan parasites such as *Trypanosoma* and *Leishmania*, where UA induces mitochondrial membrane depolarization, chromatin condensation, DNA fragmentation, activation of caspase-like proteases, and elevated reactive oxygen species (ROS) levels [38,39,40,41]. These effects lead to mitochondrial dysfunction and cell collapse, suggesting that UA can engage conserved PCD pathways analogous to those in higher eukaryotes. Notably, UA has demonstrated efficacy in both cutaneous and visceral models of leishmaniasis, underscoring its potential as a broad-spectrum antiparasitic agent. In addition to its pro-apoptotic activity, UA has recently been identified as a promising modulator of autophagy, a secondary mechanism that may contribute to parasite elimination [41]. Structural modification of UA has emerged as a critical strategy to enhance antiparasitic activity. Chemical modifications at the C-3 and C-28 positions of the ursolic acid (UA) scaffold have been particularly associated with enhanced bioactivity; for instance, ursolic and oleanolic acid derivatives esterified at these positions have demonstrated increased antileishmanial activity [42]. Similarly, other studies have reported that modifications on triterpenoid scaffolds significantly increase their effectiveness against *Leishmania* and *Trypanosoma* species, likely by improving membrane permeability and targeting key cellular pathways. Collectively, these findings support the strategic modification of the C-3 and C-28 positions in UA derivatives as a promising approach to potentiate amoebicidal activity against *N. fowleri*. Nevertheless, further studies will be required to validate the efficacy and safety of these compounds in vivo using a mouse model of *N. fowleri* infection, followed by clinical evaluation in humans.

## 4. Materials and Methods

### 4.1. General Procedures

Bruker Avance 500 and 600 spectrometers were employed for the acquisition of 1D and 2D NMR spectra (Bruker, Wissembourg, France). Chemical shifts were reported in δ (ppm), while coupling constants were expressed in Hz, with reference to the residual deuterated solvent (CDCl_3_: δ_H_ 7.26, δ_C_ 77.16), and TMS was utilized as an internal reference. ESIMS and HRESIMS analyses were conducted using an LCT Premier XE Micromass Autospec spectrometer (Waters, Barcelona, Spain). Column chromatography was performed using silica gel 60 (particle sizes 15–40 and 63–200 µm), and for analytical and preparative thin-layer chromatography (TLC), Polygram Sil G/UV254 plates (Panreac, Barcelona, Spain) were used. All reaction progress was monitored by using TLC, and visualization of spots was achieved through UV light exposure and heating of silica gel plates sprayed with H_2_O-H_2_SO_4_-AcOH (1:4:20). Unless specified otherwise, solvents and reagents were procured from commercial suppliers and used without further purification. Analytical-grade solvents from Panreac and reagents from Sigma Aldrich (St Louis, MO, USA) were employed. Ursolic acid (UA, **1**), used as starting material, was sourced from Cymit Quimica S. L. (Barcelona, Spain).

### 4.2. Plant Material

Leaves of *Mentha rotundifolia* (L.) Huds were collected in April 2021 from the northwest of Tunisia (Governorate of Béja; 36°43′36.5″ N 9°08′44.1″ E). Botanical identification was carried out by Dr. Fatima Tajini (Associate Professor, Higher Institute of Biotechnology of Béja, University of Jendouba). A voucher specimen (ISBB-MR-2021-04)) has been deposited in the Herbarium of the Higher Institute of Biotechnology of Béja, University of Jendouba (Béja, Tunisia).

### 4.3. Dried of Vegetal Material

The leaves were dried, shielded from light, and kept at room temperature (19–22 °C) for two weeks; then the plant material was ground into a fine powder using an electric herb grinder (particles size: 10-120 mesh).

### 4.4. Extracts Preparation

The air-dried and powdered leaves of *M. rotundifolia* (34.0 g) were successively extracted using an increasing polarity of solvent with hexanes (Hx, 500 mL), acetone (Ace, 500 mL), and ethanol (EtOH, 500 mL) by a Soxhlet for 24 h for each one. The organic solvents were removed under reduced pressure at 40 °C using a rotary evaporator, yielding Hx (636.6 mg), Ace (940.2 mg), and an ethanolic (EtOH, 250.1 mg) extracts. Around 10 mg from each extract was tested for its antiprotozoal activity, revealing that the hexanes extract was the most active one. The results of this preliminary study motivate us to deepen our research. For this purpose, 3 kg of *M. rotundifolia* was extracted with hexanes (3 L, 24 h) using a Soxhlet yielding 96.7 g of extract after concentration.

### 4.5. Bioactivity-Guided Chromatographic Fractionation and Isolation

The hexanes extract (96.7 g) was subjected to silica gel column chromatography, using hexanes/EtOAc mixtures of increasing polarity (from 9:1 to 0:10) as eluents to afford six fractions, which were combined based on their TLC profile in fractions M1-M4. Amoebicidal activity tests revealed that fraction M4 was active against the tested parasites. This fraction (7.1 g) was further fractioned by polarity chromatography on silica gel using hexanes/EtOAc (6:4 to 4:6) as eluent, affording six sub-fractions, which were combined based on their TLC profiles in five sub-fractions designated M4A-M4E. Sub-fractions M4B and M4C were further divided into two sub-fractions, each by precipitation using ethyl acetate as a solvent. The resulting supernates (M4B1 and M4C1) and the solid (M4B2 and M4C2) were dried and tested against the parasites, revealing that the solid sub-fractions were the most active. The active sub-fraction M4B2 was then purified by preparative thin-layer chromatography (PTLC) to yield ursolic acid. The structure characterization of the isolated compound was carried out by analysis of NMR spectroscopic data (Supporting Information, pages S2–S12), and comparison of their spectral data with those reported in the literature [24]

### 4.6. Structural Chemical Modification of Ursolic Acid

#### 4.6.1. Preparation of Derivative 2

A solution of **1** (125.0 mg, 0.26 mmol), carbonate de potassium (K_2_CO_3_, 161.0 mg, 0.86 mmol), a dimethylformamide (DMF, 3 mL), and iodomethane (CH_3_I, 70 mg, 0.49 mmol) was stirred at room temperature for 20 min. The progress of the reaction was followed by TLC using dichloromethane (DCM)/ethyl acetate (EtOAc) (9/1) as a mobile phase. After 1 h, 0.5 mL of sodium hydroxide (0.1 M) was added, and the mixture was stirred for 30 min. Then, 10 mL of water was added to the residue, and the mixture was extracted with dichloromethane (3 × 15 mL); the combined organic phases were dried over sodium sulphate and filtered off the drying agent by gravity. The solvent was removed under reduced pressure using a rotary evaporator. The residue was purified by silica gel column chromatography (CC) using an increasing polarity gradient of DCM/EtOAc from 10:0 to 10:1, yielding compound (**2**) (94 mg, 76.8%).

#### 4.6.2. General Procedure for Preparation of Derivatives 3–8

A solution of **2**, dry triethylamine, a catalytic amount of 4-(dimethylamino)-pyridine (DMAP), and an excess of the corresponding carboxylic acid derivative in dry dichloromethane (CH_2_Cl_2_) was stirred at room temperature under different sets of conditions. The progress of the reaction was monitored by TLC using hexanes/EtOAc (9/1). After the mixture was concentrated to dryness under reduced pressure, the residue was purified by silica gel CC using an increasing polarity gradient of hexanes/EtOAc (10/0 to 10:1) to afford the corresponding derivatives.

##### Preparation of Derivative **3**

A solution of **2** (20.8 mg, 0.04 mmol), triethylamine (36.4 mg, 0.36 mmol), an anhydride chloroacetic (22.0 mg, 0.13 mmol), and DMAP (5 mg) in CH_2_Cl_2_ (2 mL) was stirred for 10 min. The residue was purified by silica gel CC using increasing polarity of hexanes/EtOAc from 10:0 to 10:1, to afford the compound **3** (3.7 mg, 16.9%). 3-*O*-Chloroacetyl-methyl ursolate (**3**). [α]_D_^20^: +41.5 (c 0.33, CDCl_3_). ^1^H NMR (CDCl_3_, 600 MHz) δ: 0.74 (3H, s, Me-26), 0.86 (1H, m, H-5), 0.86 (3H, d, *J* = 6.4 Hz, Me-29), 0.87 (3H, s, Me-24), 0.88 (3H, s, Me-23), 0.94 (3H, d, *J* = 6.4Hz, Me-30), 0.95 (3H, s, Me-25), 0.99 (1H, m, H-20), 1.06 (1H, m, H-15), 1.07 (3H, s, Me-27), 1.08 (1H, m, H-1), 1.31 (1H, m, H-7), 1.32 (1H, m, H-19), 1.29 (1H, m, H-21), 1.36 (1H, m, H-6), 1.47 (1H, m, H-21), 1.48 (1H, m, H-7), 1.52 (1H, m, H-9), 1.51 (1H, m, H-6), 1.58 (1H, m, H-22), 1.60 (2H, m, H-2), 1.65 (2H, m, H-16, H-22), 1.66 (1H, m, H-1), 1.67 (1H, m, H-11), 1.78 (1H, m, H-15), 1.91 (1H, m, H-11), 1.99 (1H, m, H-16), 2.23 (1H, d, *J* = 11.4 Hz, H-18), 4.58 (1H, dd, *J* = 7.7 Hz, 9.0 Hz, H-3), 5.24 (1H, t, *J* = 3.6 Hz, H-12), chloroacetyl [4.06 (1H, d, *J* = 14.6 Hz, CH_2_), 4.09 (1H, d, *J* = 14.6 Hz, CH_2_)], 3.60 (3H, s, OCH_3_). RMN ^13^C (CDCl_3_, 150 MHz) δ: 15.7 (CH_3_ -25), 16.8 (CH_3_-24), 17.1 (CH_3_-26), 17.2 (CH_3_-29), 18.4 (CH_2_-6), 21.3 (CH_3_-30), 23.5 (CH_2_-2), 23.6 (CH_2_-11), 23.8 (CH_3_-27), 24.4 (CH_2_-16), 28.2 (CH_2_-15), 28.3 (CH_3_ -23), 30.8 (CH_2_-21), 33.1 (CH_2_-7), 36.8 (CH_2_-22), 37.1 (C-10), 38.1 (C-4), 38.4 (CH_2_-1), 39.1 (CH-20), 39.2 (CH-19), 39.7 (C-8), 42.2 (C-14), 47.7 (CH-9), 48.3 (C-17), 53.1 (CH-18), 55.5 (CH-5), 83.5 (CH-3), 125.6 (CH-12), 138.4 (C-13), 178.2 (C-28), chloroacetyl [41.4 (CH_2_), 167.3 (COO-)], 51.6 (OCH_3_). ESIMS *m/z* 569 [M + Na]^+^ (100), m/z 571 [M + Na]^+^ (39). HRESIMS *m/z* 569.3375 (calcd for C_33_H_51_O_4_NaCl, [M + Na]^+^ 569.3374), *m/z* 571.3362 (calcd for C_33_H_51_O_4_NaCl, [M + Na]^+^ 571.3368).

##### Preparation of Derivative **4**

A solution of **2** (9.9 mg, 0.02 mmol), triethylamine (Et_3_N, 36.4 mg, 0.4 mmol), a bromo-butyryl chloride (12.1 mg, 0.06 mmol), and DMAP (5 mg) in CH_2_Cl_2_ (1 mL) was stirred for 35 min. The residue was purified by silica gel CC using increasing polarity of hexanes/EtOAc (10:0.1 to 10:0.5) and by preparative thin-layer chromatographic (TLC) eluting with a mixture of hexanes/EtOAc (9/1) to afford compound **4** (3.8 mg, 30.7%). 3-*O*-(4-Bromobutyryl)-methyl ursolate (**4**). [α]_D_^20^: +26.2 (c 0.38, CDCl_3_). ^1^H NMR (CDCl_3_, 500 MHz) δ: 0.74 (3H, s, CH_3_-26), 0.85 (1H, m, H-5), 0.87 (3H, d, *J* = 6.4 Hz, CH_3_-29), 0.88 (3H, s, CH_3_-24), 0.89 (3H, s, CH_3_-23), 0.94 (3H, d, *J* = 6.3 Hz, CH_3_-30), 0.95 (3H, s, CH_3_-25), 1.03 (1H, m, H-20), 1.07 (3H, s, CH_3_-27), 1.09 (1H, m, H-15), 1.10 (1H, m, H-1), 1.32 (1H, m, H-21), 1.34 (1H, m, H-7), 1.37 (1H, m, H-19), 1.40 (1H, m, H-6), 1.51 (1H, m, H-21), 1.52 (1H, m, H-7), 1.54 (1H, m, H-6), 1.55 (1H, m, H-9), 1.62 (1H, m, H-22), 1.65 (2H, m, H-2), 1.67 (1H, m, H-1), 1.69 (1H, m, H-22), 1.70 (2H, m, H-1, H-16), 1.79 (1H, m, H-15), 1.93 (2H, m, H-11), 2.03 (1H, m, H-16), 2.23 (1H, d, *J* = 11.2 Hz, H-18), 4.52 (1H, dd, *J* = 6.5, 8.6 Hz, H-3), 5.24 (1H, t, *J* = 3.2 Hz, H-12), bromobutyryl [2.18 (2H, m), 2.51 (2H, t, *J* = 7.1 Hz), 3.47 (2H, t, *J* = 6.5 Hz)], 3.60 (3H, s, OCH_3_). ^13^C NMR (CDCl_3_, 125 MHz) δ: 15.6 (CH_3_ -25), 16.9(CH_3_-24), 17.1 (CH_3_-26), 17.2 (CH_3_-29), 18.3 (CH_2_-6), 21.3 (CH_3_-30), 23.4 (CH_2_-2), 23.7 (CH_3_-27, CH_2_-11), 24.4 (CH_2_-16), 28.2 (CH_2_-15), 28.3 (CH_3_-23), 30.8 (CH_2_-21), 33.0 (CH_2_-7), 36.8 (CH_2_-22), 37.0 (C-10), 37.9 (C-4), 38.4 (CH_2_-1), 39.0 (CH-20), 39.2 (CH-19), 39.6 (C-8), 42.1 (C-14), 47.6 (CH-9), 48.2 (C-17), 53.0 (CH-18), 55.4 (CH-5), 81.4 (CH-3), 125.6 (CH-12), 138.3 (C-13), 178.2 (C-28), bromobutyryl [33.1 (CH_2_), 32.9 (CH_2_), 28.1 (CH_2_), 172.4 (COO-)], 51.6 (OCH_3_). ESIMS *m/z* 643 [M + Na]^+^ (100%), *m/z* 641 [M + Na]^+^ (40% ). HRESIMS *m/z* 643.3169 (calcd for C_35_H_55_O_4_NaBr, [M + Na]^+^ 643.3161), *m/z* 641.3187 (calcd for C_35_H_55_O_4_NaBr, [M + Na]^+^ 641.3181).

##### Preparation of Derivative **5**

A solution of **2** (11.7 mg, 0.03 mmol), triethylamine (27.3 mg, 0.3 mmol), a 6-bromo-hexanoyl chloride (105.0 mg, 0.46 mmol), and DMAP (5 mg) in CH_2_Cl_2_ (2 mL) was stirred overnight. The residue was purified by silica gel CC using increasing polarity of hexanes/EtOAc (10:0.1 to 10:0.5), affording the compound **5** (5.1 mg, 26.2%). 3-*O*-(6-Bromo-hexanoyl)-methyl ursolate (**5**). [α]_D_^20^: +36.9 (c 0.51, CDCl_3_). ^1^H NMR (CDCl_3_, 600 MHz) δ: 0.74 (3H, s, CH_3_-26), 0.87 (3H, d, *J* = 6.4 Hz, CH_3_-29), 0.83 (1H, m, H-5), 0.86 (3H, s, CH_3_-24), 0.87 (3H, s, CH_3_-23), 0.94 (3H, d, *J* = 6.3 Hz, CH_3_-30), 0.95 (3H, s, CH_3_-25), 1.01 (1H, m, H-20), 1.07 (3H, s, CH_3_-27), 1.08 (1H, m, H-15), 1.08 (1H, m, H-1), 1.30 (1H, m, H-21), 1.32 (1H, m, H-7), 1.34 (1H, m, H-19), 1.37 (1H, m, H-6), 1.48 (1H, m, H-21), 1.49 (1H, m, H-7), 1.52 (1H, m, H-6), 1.36 (1H, m, H-9), 1.60 (1H, m, H-22), 1.67 (1H, m, H-1), 1.67 (1H, m, H-22), 1.68 (2H, m, H-1, H-16), 1.91(2H, m, H-11), 1.79 (1H, m, H-15), 1.64 (2H, m, H-2), 2.00 (1H, m, H-16), 2.23 (1H, d, *J* = 11.2 Hz, H-18), 4.50 (1H, t, *J* = 6.2 Hz, H-3), 5.24 (1H, t, *J* = 3.1 Hz, H-12), bromohexanoyl [1.48 (2H, m), 1.68 (2H, m), 1.88 (2H, m), 2.32 (2H, t, *J* = 7.6 Hz), 3.40 (2H, t, *J* = 6.7 Hz)]. 3.60 (3H, s, OCH_3_). ^13^C NMR (CDCl_3_, 150 MHz) δ: 15.7 (CH_3_ -25), 17.0 (CH_3_-24), 17.1 (CH_3_-26), 17.2 (CH_3_-29), 18.4 (CH_2_-6), 21.3 (CH_3_-30), 23.5 (CH_2_-2), 23.7 (CH_3_-27), 23.8 (CH_2_-11), 24.4 (CH_2_-16), 27.9 (CH_2_-15), 28.3 (CH_3_-23), 30.8 (CH_2_-21), 33.1 (CH_2_-7), 36.8 (CH_2_-22), 37.0 (C-10), 37.9 (C-4), 38.4 (CH_2_-1), 39.0 (CH-20), 39.2 (CH-19), 39.7 (C-8), 42.2 (C-14), 47.7 (CH-9), 48.3 (C-17), 53.1 (CH-18), 55.5 (CH-5), 81.0 (CH-3), 125.6 (CH-12), 138.4 (C-13), 178.2 (C-28), bromohexanoyl [34.7 (CH_2_), 33.6 (CH_2_), 32.6 (CH_2_), 28.2 (CH_2_), 24.4 (CH_2_), 173.4 (COO-)], 51.6 (OCH_3_). ESIMS *m/z* 671 [M + Na]^+^ (100), *m/z* 669 [M + Na]^+^ (38). HRESIMS *m/z* 671.3472 (calcd for C_37_H_59_O_4_NaBr, [M + Na]^+^ 671.3474), *m/z* 669.3488 (calcd for C_37_H_59_O_4_NaBr, [M + Na]^+^ 669.3494).

##### Preparation of Derivative **6**

A solution of **2** (12.8 mg, 0.03 mmol), 4-bromomethyl benzoic acid (20.0 mg, 0,09 mmol), DMF (0.5 mL), and DMAP (5 mg) with *N*,*N*-dicyclohexylcarbodiimide (DCC) in CH_2_Cl_2_ (1 mL) was stirred for 38 h. The residue was purified by silica gel CC using increasing polarity of hexanes/EtOAc from 10:0.1 to 10:0.5, to afford compound **6** (1.2 mg, 6.0%). 3-*O*-(4-Bromomethylbenzoyl)-methyl ursolate (6). [α]_D_^20^: + 49.2 (c 0.12, CDCl_3_). ^1^H NMR (CDCl_3_, 500 MHz) δ: 0.76 (3H, s, CH_3_-26), 0.87 (3H, d, *J* = 6.4 Hz, CH_3_-29), 0.91 (1H, m, H-5), 0.93 (3H, s, CH_3_-23), 0.95 (3H, d, *J* = 6.3 Hz, CH_3_-30), 0.99 (3H, s, CH_3_-25), 1.00 (3H, s, CH_3_-24), 1.01 (1H, m, H-20), 1.08 (1H, m, H-15), 1.09 (3H, s, CH_3_-27), 1.16 (1H, m, H-1), 1.32 (1H, m, H-21), 1.34 (1H, m, H-7), 1.35 (1H, m, H-19), 1.42 (1H, m, H-6), 1.49 (1H, m, H-21), 1.53 (1H, m, H-7), 1.56 (1H, m, H-6), 1.57 (1H, m, H-9), 1.60 (1H, m, H-22), 1.67 (1H, m, H-22), 1.68 (2H, m, H-1, H-16), 1.70 (1H, m, H-1), 1.77 (2H, m, H-2), 1.80 (1H, m, H-15), 1.94 (2H, m, H-11), 2.02 (1H, m, H-16), 2.26 (1H, d, *J* = 11.6 Hz, H-18), 4.73 (1H, dd, *J* = 6.5, 8.6 Hz, H-3), 5.26 (1H, t, *J* = 3.2 Hz, H-12), bromomethylbenzoyl [4.50 (2H, s), 7.45 (2H, d, *J* = 8.2 Hz), 8.01 (2H, d, *J* = 8.2 Hz)], 3.61 (3H, s, OCH_3_). ^13^C NMR (CDCl_3_, 125 MHz) δ: 14.3 (CH_3_-25), 15.7 (CH_3_-24), 17.1 (CH_3_-26), 17.2 (CH_3_-29), 18.4 (CH_2_-6), 21.3 (CH_3_-30), 23.5 (CH_2_-2), 23.8 (CH_3_-27, CH_2_-11), 24.4 (CH_2_-16), 28.2 (CH_2_-15), 28.4 (CH_3_-23), 30.8 (CH_2_-21), 33.1 (CH_2_-7), 36.8 (CH_2_-22), 37.1 (C-10), 38.2 (C-4), 38.5 (CH_2_-1), 39.0 (CH-20), 39.2 (CH-19), 39.7 (C-8), 42.2 (C-14), 47.7 (CH-9), 48.3 (C-17), 53.1 (CH-18), 55.5 (CH-5), 82.0 (CH-3), 125.6 (CH-12), 138.4 (C-13), 178.2 (C-28), bromomethylbenzoyl [32.4 (CH_2_), 129.1 (2 × CH), 130.2 (2 x CH), 131.1 (C), 142.6 (C), 165.9 (COO-)], 51.6 (OCH_3_). ESIMS *m/z* 689 [M + Na]^+^ (100), 691 [M + Na]^+^ (38). HRESIMS *m/z* 689.3186 (calcd for C_39_H_55_O_4_NaBr, [M + Na]^+^ 689.3181), *m/z* 691.3178 (calcd for C_39_H_55_O_4_NaBr, [M + Na]^+^ 691.3161).

##### Preparation of Derivative **7**

A solution of **2** (8.1 mg, 0.02 mmol), triethylamine (9.1 mg, 0.2 mmol), succinic anhydride (10.0 mg, 0.08 mmol), and DMAP (5 mg) in DMF (0.5 mL) was stirred for 30 min. Subsequently, acetone (1 mL) and concentrated hydrochloric acid (HCl, 0.01 mL, 1M) were added, and the mixture was stirred for 30 min. After removing the solvent, the residue was purified by silica gel column CC using an increasing polarity of hexanes/EtOAc from 8:2 to 5:5 to afford compound **7** (3.0 mg, 26.3%). 3-*O*-(Hemisuccinyl)-methyl ursolate (**7**). [α]_D_^20^: +33.7 (c 0.3, CH_3_OH). RMN ^1^H (CD_3_OD, 500 MHz) δ: 0.78 (3H, s, CH_3_-26), 0.87 (1H, m, H-5), 0.89 (3H, d, *J* = 6.4 Hz, CH_3_-29), 0.89 (3H, s, CH_3_-24), 0.90 (3H, s, CH_3_-23), 0.98 (3H, d, *J* = 6.4 Hz, CH_3_-30), 0.99 (3H, s, CH_3_-25), 1.01 (1H, m, H-20), 1.06 (1H, m, H-1), 1.09 (1H, m, H-15), 1.3 (3H, s, CH_3_-27), 1.34 (1H, m, H-7), 1.36 (1H, m, H-21), 1.41 (1H, m, H-19), 1.43 (1H, m, H-6), 1.50 (1H, m, H-21), 1.56 (1H, m, H-6, H-7), 1.61 (1H, m, H-9), 1.65 (1H, m, H-22), 1.65 (2H, m, H-2), 1.67 (1H, m, H-16), 1.69 (1H, m, H-1), 1.81 (1H, m, H-15), 1.94 (2H, m, H-11), 2.07 (1H, m, H-16), 2.23 (1H, d, *J* = 11.2 Hz, H-18), 4.49 (1H, dd, *J* = 4.3, 8.6 Hz, H-3), 5.24 (1H, t, *J* = 2.9 Hz, H-12), hemisuccinyl [2.85 (4H, m)], 3.60 (3H, s, OCH_3_). RMN ^13^C (CD_3_OD, 125 MHz) δ: 16.0 (CH_3_ -25), 17.3 (CH_3_-24), 17.6 (CH_3_-26), 17.7 (CH_3_-29), 19.3 (CH_2_-6), 21.5 (CH_3_-30), 24.1 (CH_2_-27), 24.3 (CH_2_-2), 24.5 (CH_2_-11), 25.3 (CH_2_-16), 28.6 (CH_3_-23), 29.1 (CH_2_-15), 30.7 (CH_2_-21), 34.1 (CH_2_-7), 37.9 (CH_2_-22), 38.0 (C-10), 38.8 (C-4), 39.3 (CH-20), 39.4 (CH_2_-1), 39.4 (CH-19), 40.8 (C-8), 43.2 (C-14), 48.9 (C-17), 49.4 (CH-9), 54.4 (CH-18), 56.7 (CH-5), 82.7 (CH-3), 127.0 (CH-12), 139.6 (C-13), 179.9 (C-28), hemisuccinyl [31.6 (CH_2_), 30.2 (CH_2_), 174.2 (COO-), 176.2 (COOH)], 52.1 (OCH_3_). ESIMS *m/z* 569 [M-1]^+^ (100). HRESIMS *m/z* 569.3851 (calcd for C_35_H_53_O_6_, [M-1]^+^ 569.3842).

##### Preparation of Derivative **8**

A solution of **2** (7.3 mg, 0.02 mmol), triethylamine (9.1 mg, 0.02 mmol), glutaric anhydride (11.1 mg, 0.12 mmol), and DMAP (5 mg) in DMF (0.5 mL) was stirred at room temperature for 30 min. Subsequently, acetone (1 mL) and HCL (0.01 mL, 1M) were added, and the mixture was stirred for 2 h. The solvent was removed under reduced pressure using a rotary evaporator, and the residue was purified by silica gel column chromatography (CC) using an increasing polarity of hexanes/EtOAc (8:2 to 5:5) to afford compound **8** (1.1 mg, 9.4%). 3-*O*-(Hemiglutaryl)-methyl ursolate (**8**). [α]_D_^20^: +74.5 (c 0.11, CH_3_OH). ^1^H NMR (CD_3_OD, 500 MHz) δ: 0.78 (3H, s, CH_3_-26), 0.86 (1H, m, H-5), 0.88 (6H, CH_3_-29, CH_3_-24), 0.89 (3H, s, CH_3_-23), 0.97 (3H, d, *J* = 6.3 Hz, CH_3_-30), 1.00 (3H, s, CH_3_-25), 0.99 (1H, m, H-20), 1.13 (3H, s, CH_3_-27), 1.09 (1H, m, H-15), 1.07 (1H, m, H-1), 1.35 (1H, m, H-21), 1.34 (1H, m, H-7), 1.39 (1H, m, H-19), 1.44 (1H, m, H-6), 1.50 (1H, m, H-21), 1.56 (1H, m, H-7), 1.56 (1H, m, H-6), 1.60 (1H, m, H-9), 1.58 (1H, m, H-22), 1.63 (2H, m, H-2), 1.69 (1H, m, H-1), 1.65 (1H, m, H-22), 1.67 (1H, m, H-1, H-16), 1.81 (1H, m, H-15), 1.94 (2H, m, H-11), 2.07 (1H, m, H-16), 2.23 (1H, d, *J* = 11.2 Hz, H-18), 4.49 (1H, dd, *J* = 4.2, 10.0 Hz, H-3), 5.24 (1H, t, *J* = 2.7 Hz, H-12), hemiglutaryl [ 2.58 (4H, s), 1.79 (2H, m)], 3.60 (3H, s, OCH_3_). ^13^C NMR (CD_3_OD, 125 MHz) δ: 16.0 (CH_3_ -25), 17.2 (CH_3_-24), 17.6 (CH_3_-26), 17.7 (CH_3_-29), 19.3 (CH_2_-6), 21.5 (CH_3_-30), 24.1 (CH_3_-27), 24.3 (CH_2_-2), 24.5 (CH_2_-11), 25.3 (CH_2_-16), 28.6 (CH_3_-23), 29.1 (CH_2_-15), 31.6 (CH_2_-21), 34.1 (CH_2_-7), 37.8 (CH_2_-22), 38.0 (C-10), 38.8 (C-4), 39.4 (CH_2_-1), 40.3 (CH-20), 40.4 (CH-19), 40.8 (C-8), 43.2 (C-14), 48.0 (C-17), 48.8 (CH-9), 54.4 (CH-18), 56.8 (CH-5), 82.6 (CH-3), 127.0 (CH-12), 139.6 (C-13), 179.8 (C-28), hemiglutaryl [30.3 (CH_2_), 30.8 (2 × CH_2_), 174.2 (COO-), 175.0 (COOH)]. 52.1 (OCH_3_). ESIMS *m/z* 583 [M-1]^+^ (100). HRESIMS *m/z* 583.3991 (calcd for C_36_H_55_O_6_, [M-1]^+^ 583.3999).

#### 4.6.3. General Procedure for Preparation of Derivatives 9–11

A solution of *3-O-chloroacetyl*-*methyl ursolate* (**3**), a catalytic amount of potassium fluoride (KF) in acetonitrile (CH_3_CN), and an excess of the corresponding nitrogen compounds were stirred at room temperature. The progress of the reaction was monitored by TLC using hexanes/EtOAc (9/1) or EtOAc/MeOH (9/1). After the mixture was concentrated to dryness under reduced pressure, the residue was purified by a silica gel CC to afford the corresponding derivatives.

##### Preparation of Derivative **9**

A solution of **3** (10.6 mg, 0.02 mmol), sodium azide (NaN_3,_ 13.0 mg, 0,2 mmol), and KF (4.0 mg, 0.07 mmol) in CH_3_CN (1 mL) was stirred for 50 min. The residue was purified by silica gel CC using an increasing polarity of hexanes/EtOAc from 10:0.5 to 10:1, yielding compound **9** (7.4 mg, 66.8%). 3-*O*-(Azidoacetyl)-methyl ursolate (**9**). [α]_D_^20^: + 49.5 (c 0.74, CDCl_3_). ^1^H NMR (CDCl_3_, 600 MHz) δ: 0.75 (3H, s, CH_3_-26), 0.86 (3H, d, *J* = 6.4 Hz, CH_3_-29), 0.87 (1H, m, H-5), 0.88 (3H, s, CH_3_-24), 0.89 (3H, s, CH_3_-23), 0.94 (3H, d, *J* = 6.3 Hz, CH_3_-30), 0.95 (3H, s, CH_3_-25), 1.34 (1H, m, H-19), 1.08 (3H, s, CH_3_-27), 1.09 (1H, m, H-15), 1.12 (1H, m, H-1), 1.32 (1H, m, H-21), 1.35 (1H, m, H-7), 1.01 (1H, m, H-20), 1.41 (1H, m, H-6), 1.51 (1H, m, H-21), 1.53 (1H, m, H-7), 1.55 (1H, m, H-6), 1.56 (1H, m, H-9), 1.62 (1H, m, H-22), 1.69 (1H, m, H-22), 1.70 (2H, m, H-1, H-16), 1.71 (2H, m, H-11), 1.80 (1H, m, H-15), 1.94 (2H, m, H-2), 2.03 (1H, m, H-16), 2.23 (1H, d, *J* = 11.2 Hz, H-18), 4.62 (1H, t, *J* = 7.7 Hz, H-3), 5.24 (1H, t, *J* = 3.5 Hz, H-12), azidoacetyl [3.85 (2H, s)], 3.60 (3H, s OCH_3_). ^13^C NMR (CDCl_3_, 150 MHz) δ: 15.6 (CH_3_ -25), 16.9 (CH_3_-24), 17.1 (CH_3_-26), 17.2 (CH_3_-29), 18.4 (CH_2_-6), 21.3 (CH_3_-30), 23.5 (CH_2_-2), 23.7 (CH_3_-27), 23.7 (CH_2_-11), 24.4 (CH_2_-16), 28.2 (CH_2_-15), 28.3 (CH_3_-23), 30.8 (CH_2_-21), 33.0 (CH_2_-7), 36.8 (CH_2_-22), 37.0 (C-10), 37.9 (C-4), 38.4 (CH_2_-1), 39.0 (CH-20), 39.2 (CH-19), 39.7 (C-8), 42.2 (C-14), 47.6 (CH-9), 48.3 (C-17), 53.0 (CH-18), 55.5 (CH-5), 83.4 (CH-3), 125.5 (CH-12), 138.4 (C-13), 178.2 (C-28), azidoacetyl [50.8 (CH_2_), 168.3 (COO-)], 51.6 (OCH_3_). ESIMS *m/z* 576 [M + Na]^+^ (100). HRESIMS *m/z* 576.3785 (calcd for C_33_H_51_N_3_O_4_Na, [M + Na]^+^ 576.3777).

##### Preparation of Derivative **10**

A solution of **3** (10.1 mg, 0.02 mmol), *N*-methyl-piperazin (45.2 mg, 0.4 mmol), and KF (4.0 mg, 0.07 mmol) in CH_3_CN (1mL) was stirred overnight. The residue was purified by silica gel CC using increasing polarity from EtOAc/MeOH (8:2) to MeOH yielding compound **10** (11.0 mg, 90%). 3-*O*-(Methylpiperazin-acetyl)-methyl ursolate (**10**). [α]_D_^20^: +25.8 (c 1.1, CH_3_OH). ^1^H NMR (CD_3_OD, 500 MHz) δ: 0.78 (3H, s, CH_3_-26), 0.86 (1H, m, H-5), 0.89 (9H, CH_3_-23, CH_3_-24, CH_3_-29), 0.98 (3H, d, *J* = 6.3 Hz, CH_3_-30), 0.99 (1H, m, H-20), 1.00 (3H, s, CH_3_-25), 1.08 (1H, m, H-1), 1.10 (1H, m, H-15), 1.13 (3H, s, CH_3_-27), 1.34 (1H, m, H-21), 1.35 (1H, m, H-7), 1.40 (1H, m, H-19), 1.44 (1H, m, H-6), 1.48 (1H, m, H-7), 1.49 (1H, m, H-21), 1.56 (2H, m, H-6, H-7), 1.59 (1H, m, H-22), 1.60 (1H, m, H-9), 1.64 (1H, m, H-2), 1.66 (2H, m, H-2, H-22), 1.67 (1H, m, H-1, H-16), 1.70 (1H, m, H-1), 1.81 (1H, m, H-15), 1.94 (2H, m, H-11), 2.07 (1H, m, H-16), 2.23 (1H, d, *J* = 11.2 Hz, H-18), 4.55 (1H, dd, *J* = 4.5, 11.2 Hz, H-3), 5.24 (1H, t, *J* = 3.2 Hz, H-12), methylpiperazin-acetyl [2.28 (3H, s), 2.52 (2H, s), 2.64 (2H, s), 3.25 (2H, s)], 3.60 (3H, s, OCH_3_). ^13^C NMR (CD_3_OD, 125 MHz) δ: 16.0 (CH_3_ -25), 17.3 (CH_3_-24), 17.6 (CH_3_-26, CH_3_-29), 19.3 (CH2-6), 21.5 (CH3-30), 24.2 (CH_3_-27), 24.3 (CH_2_-2), 24.6 (CH_2_-11), 25.3 (CH_2_-16), 28.7 (CH_3_-23), 29.1 (CH_2_-15), 31.6 (CH_2_-21), 34.1 (CH_2_-7), 37.9 (C-10), 38.8 (C-4), 38.0 (CH_2_-22), 39.4 (CH_2_-1), 40.3 (CH-20, CH-19), 40.8 (C-8), 43.2 (C-14), 49.0 (CH-9, C-17), 54.4 (CH-18), 56.7 (CH-5), 82.9 (CH-3), 126.9 (CH-12), 139.6 (C-13), 179.8 (C-28), methylpiperazin-acetyl [45.9 (CH_3_), 55.3 (2 x CH_2_), 55.5 (2 x CH_2_), 59.8 (CH_2_), 171.5 (COO-)], 52.1 (OCH_3_). ESIMS *m/z* 611 [M + 1]+ (100). HRESIMS *m/z* 611.4781 (calcd for C_38_H_63_N_2_O_4_, [M + 1]^+^ 611.4788).

##### Preparation of Derivative **11**

A solution of **3** (10.0 mg, 0.02 mmol), imidazole (12.0 mg, 0.18 mmol), and KF (4.0 mg, 0.07 mmol) in CH_3_CN (1mL) was stirred for 1.5 h. The residue was purified by Sephadex LH-20 eluting with CHCl_3_/MeOH 1:1, yielding compound **11** (10.0 mg, 86.4%). 3-*O*-(Imidazolin-acetyl)-methyl ursolate (**11**). [α]_D_^20^: +29.1 (c 1.0, CH_3_OH).^1^H-NMR (CD_3_OD, 500 MHz) δ: 0.77 (3H, s, CH_3_-26), 0.79 (3H, s, CH_3_-24), 0.86 (4H, s, H-5, CH3-23), 0.88 (3H, d, *J* = 6.6 Hz CH_3_-29), 0.97 (3H, d, *J* = 6.6 Hz, CH_3_-30), 0.99 (1H, m, H-20), 0.98 (3H, s, CH_3_-25), 1.06 (1H, m, H-1), 1.09 (1H, m, H-15), 1.12 (3H, s, CH_3_-27), 1.33 (1H, m, H-7), 1.35 (1H, m, H-21), 1.39 (1H, m, H-19), 1.41 (1H, m, H-6), 1.51 (1H, m, H-21), 1.55 (2H, m, H-6, H-7), 1.58 (1H, m, H-22), 1.59 (1H, m, H-9), 1.65 (2H, m, H-2, H-22), 1.67 (1H, m, H-1, H-16), 1.68 (1H, m, H-2), 1.69 (1H, m, H-1), 1.80 (1H, m, H-15), 1.93 (2H, m, H-11), 2.07 (1H, m, H-16), 2.22 (1H, d, *J* = 11.3 Hz, H-18), 4.53 (1H, t, *J* = 9.0 Hz, H-3), 5.23 (1H, t, *J* = 3.1 Hz, H-12), imidazolin-acetyl [3.62 (2H, s), 7.00 (1H, s), 7.13 (1H, s), 7.69 (1H, s)], 3.59 (3H, s, OCH_3_). ^13^C-NMR (CD_3_OD, 125 MHz) δ: 16.00 (CH_3_-25), 17.0 (CH_3_-24), 17.6 (CH_3_-26, CH_3_-29), 19.2 (CH_2_-6), 21.5 (CH_3_-30), 24.2 (CH_3_-27), 24.3 (CH_2_-2), 24.4 (CH_2_-11), 25.3 (CH_2_-16), 28.5 (CH_3_-23), 29.1 (CH_2_-15), 31.6 (CH_2_-21), 34.0 (CH_2_-7), 37.8 (CH_2_-22), 38.0 (C-10), 38.8 (C-4), 39.3 (CH_2_-1), 40.3 (CH-20, CH-19), 40.8 (C-8), 43.2 (C-14), 48.7 (CH-9, C-17), 54.3 (CH-18), 56.6 (CH-5), 84.2 (CH-3), 126.9 (CH-12), 179.9 (C-28), imidazolin-acetyl [58.3 (CH_2_), 122.0 (CH), 128.9 (CH), 133.8 (CH), (169.5 (COO-)], 52.2 (OCH_3_). ESIMS *m/z* 579 [M + 1]^+^ (40). HRESIMS *m/z* 579.4161 (calcd for C_36_H_55_N_2_O_4_, [M + 1]^+^ 579.4162).

### 4.7. Biological Activity

#### 4.7.1. Amoebic Strains and Cell Maintenance

The in vitro activity of the tested compounds against *Naegleria fowleri* trophozoites was evaluated using two clinical strains obtained from the American Type Culture Collection (ATCC^®^ 30808™ and ATCC^®^ 30215™). Trophozoites were cultured axenically at 37 °C in 2% (*w/v*) Bactocasitone medium (Thermo Fisher Scientific, Madrid, Spain), supplemented with 10% (*v/v*) fetal bovine serum (FBS), 0.3 μg/mL penicillin G sodium salt, and 0.5 mg/mL streptomycin sulfate (Sigma-Aldrich, Madrid, Spain). For cytotoxicity assessment, murine macrophages from the J774A.1 cell line (ATCC^®^ TIB-67) were cultured in Dulbecco’s Modified Eagle’s Medium (DMEM) supplemented with 10% (*v/v*) FBS and 10 μg/mL gentamicin sulfate (Sigma-Aldrich, Madrid, Spain). The macrophages were maintained at 37 °C in a 5% CO_2_ atmosphere.

#### 4.7.2. In Vitro Amoebicidal Activity Against *N. fowleri* Trophozoites

The trophocidal activity of the compounds was evaluated using the alamarBlue^®^ colorimetric assay [43]. *N. fowleri* trophozoites were seeded in duplicate in a 96-well microtiter plate at a concentration of 2 × 10^5^ cells/mL and incubated for adhesion in Bactocasitone medium (2% *v/w*) (Thermo Fisher Scientific, Madrid, Spain), supplemented with 10% (*v/v*) fetal bovine serum (FBS), 0.3% penicillin G sodium salt, and 0.5 mg/mL streptomycin sulfate (Sigma-Aldrich, Madrid, Spain). Different concentrations of the evaluated compounds, prepared in fresh Bactocasitone medium, were added to the wells (50 µL per well). The negative control consisted of trophozoites incubated with the medium alone. After adding 10 µL of alamarBlue^®^ reagent, the plates were incubated at 37 °C for 48 h with slight agitation. The data were analyzed using nonlinear regression with GraphPad Prism 9 software to determine the IC_50_ and IC_90_ values, which were then used for further studies on PCD [10].

#### 4.7.3. In Vitro Amoebicidal Activity Against *N. fowleri* Cyst

Following the assays against trophozoites, the compounds were further evaluated for their activity against the cyst stage of *N. fowleri* (ATCC^®^ 30808™ and ATCC^®^ 30215™). To induce cyst formation, the trophozoites were centrifuged, resuspended in MYAS medium, and incubated under slight agitation at room temperature for 10 days. After this, mature cysts were harvested, pretreated with 0.5% SDS, and seeded in a 96-well microtiter plate at 2 × 10^5^ cells/mL. The drugs were tested in serial dilutions, and after 24 h of incubation at 37 °C, the medium was replaced with fresh Bactocasitone medium. The colorimetric alamarBlue^®^ assay was used to assess cyst viability, with 10 µL of reagent added to each well. The plates were incubated for an additional 72 h at 37 °C, and fluorescence was measured using the EnSpire^®^ Multimode Plate Reader (PerkinElmer, Madrid, Spain). The results were compared to a negative control (untreated cysts) and a positive control (amphotericin B and miltefosine) [30]. The IC_50_ values were determined through nonlinear regression analysis.

#### 4.7.4. In Vitro Cytotoxicity on Murine Macrophage

The cytotoxicity of the compounds was assessed against the murine macrophage cell line J774A.1 (ATCC^®^ TIB-67™). Cells were maintained at 37 °C in a 5% CO_2_ atmosphere in DMEM medium. In the assay, different concentrations of the compounds were added to the cells in a 96-well microtiter plate, and after 24 h of incubation, 10 µL of alamarBlue^®^ reagent was added. The fluorescence intensity was measured using the EnSpire^®^ Multimode Plate Reader (PerkinElmer, Madrid, Spain). The cytotoxic concentration required to reduce cell viability by 50% (CC_50_) was determined through nonlinear regression analysis using GraphPad Prism 9 software. The results were compared with those obtained against trophozoites to calculate the selectivity index (S.I.) for *N. fowleri* [10].

### 4.8. Mechanism of Action on N. fowleri Trophozoites for Compounds 7 and 10

#### 4.8.1. Analysis of Programmed Cell Death

To evaluate the induction of PCD, *N. fowleri* strains ATCC^®^ 30808™ and ATCC^®^ 30215™ were treated with the IC_90_ concentration of the compounds **7** and **10** for 24 h at 37 °C. After incubation, specific fluorescent markers were used to assess various detectable metabolic events, following the manufacturers’ instructions for each assay. Amphotericin B was used as a positive control, and untreated amoebae served as a negative control. Fluorescence imaging was performed using EVOS M5000 Cell Imaging System (Life Technologies, Madrid, Spain) and confocal microscope Leica DMI 4000 B equipped with Leica Application Suite X software version 3.5.5.19976 and Leica HC PL APO 63x/1.40 OIL CS2 objective (Leica Microsystems, Wetzlar, Germany). The fluorescence obtained was expressed as mean ± standard deviation from at least three independent experiments.

#### 4.8.2. Analysis of Chromatin Condensation

To evaluate the chromatin condensation, cells were stained with Hoechst 33,342 (1 µM) and propidium iodide (PI) (1 µM) for 20 min in the dark. Hoechst 33,342 binds to condensed chromatin in apoptotic cells and emits blue fluorescence, while PI enters cells with compromised membranes, indicative of late apoptosis or necrosis, and binds to DNA, emitting red fluorescence. This dual-staining approach allowed the differentiation of three cell populations: viable cells, which emitted faint blue fluorescence (Hoechst 33,342); apoptotic cells, characterized by bright blue fluorescence indicating chromatin condensation; and dead cells, which displayed red fluorescence (PI) due to membrane disruption. Untreated cells were included as negative controls to establish baseline fluorescence levels [30].

#### 4.8.3. Analysis of Plasma Membrane Permeability

After treatment, SYTOX™ Green nucleic acid stain (Life Technologies, Madrid, Spain) was added to each well at a final concentration of 1 μg/mL and incubated for 15 min in the dark. SYTOX™ Green selectively penetrates cells with damaged membranes, binding to nucleic acids and emitting bright green fluorescence. In contrast, viable cells with intact membranes exclude the dye and remain non-fluorescent. Untreated trophozoites were used as negative controls [10].

#### 4.8.4. Analysis of Mitochondrial Membrane Potential

Mitochondrial membrane potential (ΔΨm) was assessed using the JC-1 dye (1,1′,3,3′-tetraethyl-5,5′,6,6′-tetrachloroimidacarbocyanine iodide; Cayman Chemical, Madrid); following treatment, cells were harvested by centrifugation at 2500 rpm for 10 min and resuspended in JC-1 assay buffer. A total of 10 μL of JC-1 dye was added to each well, and the plates were incubated in the dark for 20-30 min. The JC-1 dye accumulates in mitochondria in a potential-dependent manner. In healthy cells, with intact mitochondrial membrane potential (ΔΨm), JC-1 forms red fluorescent J-aggregates (emission at ~595 nm). In cells with depolarized mitochondria, JC-1 remains in its monomeric form, emitting green fluorescence (emission at ~535 nm), indicating mitochondrial damage and a loss of ΔΨm [10].

#### 4.8.5. Measurement of Intracellular Adenosine Triphosphate Levels

Adenosine triphosphate (ATP) production was quantified using the CellTiter-Glo^®^ luminescent cell viability assay (Promega, Madrid, Spain). Amoebae were treated with the compounds, and an equal volume of reagent was added per well. Following cell lysis, the emitted luminescence, which is directly proportional to ATP content, was measured using the EnSpire^®^ Multimode Plate Reader (PerkinElmer, Madrid, Spain). This assay allowed the comparison of ATP levels between treated and untreated (negative control) cells, providing insights into the impact of treatment on cellular energy metabolism [30].

#### 4.8.6. Measurement of Intracellular Reactive Oxygen Species Levels

The oxidative stress induced by the generation of ROS in *N. fowleri* was evaluated using the CellROX^®^ Deep Red reagent (Invitrogen, Thermo Fisher Scientific, Madrid, Spain). Following incubation, cells were stained with the dye at a final concentration of 5 μM and then incubated for an additional 30 min. The reagent enters cells in response to elevated ROS levels, emitting red fluorescence at approximately 644/665 nm. Increased fluorescence intensity indicated higher levels of ROS accumulation, reflecting elevated oxidative stress in the treated cells [30].

#### 4.8.7. Detection of Autophagic Vacuole Formation

Autophagy is a vital cellular process involved in the degradation and recycling of intracellular components, often activated in response to drug exposure. To evaluate autophagic activity, trophozoites were stained with monodansylcadaverine (MDC) (Sigma-Aldrich, St. Louis, MO, USA) during 30 min in the dark. This is a fluorescent dye that selectively accumulates in autophagic vacuoles due to its ion-trapping properties and affinity for membrane lipids. After treatment, cells were incubated with MDC. The dye emits a characteristic cyan-blue fluorescence, allowing the detection and qualitative analysis of autophagic vacuoles in treated cells [32].

#### 4.8.8. Analysis of Cytoskeletal Integrity

Cytoskeletal disruption in treated amoebae was evaluated by staining with phalloidin conjugated to tetramethylrhodamine B isothiocyanate (phalloidin- TRITC), which binds specifically to filamentous actin, emitting red fluorescence. DAPI staining was used in parallel to visualize nuclear DNA, emitting blue fluorescence. After treatment, cells were fixed with formaldehyde on coverslips, permeabilized with 0.1% Triton X-100, and incubated with phalloidin-TRITC (Sigma-Aldrich, St. Louis, MO, USA) for 30 min. Following washing with PBS, fluorescence images were captured with a 63x oil immersion objective [44].

#### 4.8.9. Statistical Analysis

All in vitro experiments were conducted across three independent assays (*n* = 3) under optimal growth conditions. The results are expressed as the mean ± standard deviation (SD) of triplicate assays. Statistical analysis was performed using one-way analysis of variance (ANOVA) to compare means, inhibition curves, and data graphs generated with GraphPad Prism 9.0 software (GraphPad Software; CA, USA). A *p*-value < 0.05 was considered statistically significant. Additionally, the bar graphs presented show the proportion of stained cells relative to the total amoebae population. The percentage of stained cells was quantified using FIJI software (version 1.53q; National Institutes of Health, USA) from three representative images per experimental condition for each mechanism of action.

## 5. Conclusions

Ursolic acid (UA) was identified as an amoebicidal agent against *N. fowleri* by bioassay-guided isolation of *M. rotundifolia* leaves. Subsequently, according to rational drug design strategy, a total of 10 derivatives were synthesized, including 7 previously undescribed compounds. Notably, some UA derivatives displayed potent amoebicidal activity in a micromolar range, outperforming both the UA and the reference drug, miltefosine. These findings underscore the significance of structural modifications at the C-3 and C-28 positions on the ursane-type triterpenoid skeleton. Based on pharmacological mechanism research, selected compounds **7** and **10** induce programmed cell death via apoptosis- and autophagy-like mechanisms, along with marked disruption of the actin cytoskeleton, suggesting that these compounds act through multiple, complementary pathways. Taken together, these findings provide strong evidence for the therapeutic potential of UA-based derivatives as multifunctional agents capable of targeting essential cellular processes in *N. fowleri*, meriting further investigation for its potential development as an anti-infective agent against Primary Amoebic Meningoencephalitis.

## Figures and Tables

**Table 1:** Antiparasitic activity against *N. fowleri* trophozoites of extracts, fractions, subfractions, and isolated compound from *M. rotundifolia* leaves.

Samples	*N. fowleri* ATCC^®^ 30808^TM^ IC_50_ ^a^ µg/mL
Hx ^b^	134.59 ± 14.57
Hx ^c^	129.38 ± 7.18
M4	76.28 ± 2.39
M4A	122.5 ± 6.51
M4B1	54.75 ± 8.56
M4B2	65.11 ± 8.08
M4C1	96.91 ± 8.46
M4C2	74.11 ± 10.18
M4D	94.35 ± 10.39
M4E	77.6 ± 0.85
UA (1) ^d^	65.91 ± 3.73
M ^e^	12.73 ± 1.49
A ^e^	0.162 ± 0.002

^a^ IC_50_: Concentrations able to inhibit 50% of the growth of the tested parasite, expressed in µg/mL ± standard deviation (SD). ^b^ Hx: Hexanes extract from polarity gradient extraction, ^c^ Hx: Hexanes extract from bio-guided fractionation, ^d^ UA (**1**): Ursolic acid, ^e^ M: Miltefosine, and A: Amphotericin B were used as positive controls. Antiparasitic activities were performed as independent experiments in triplicate. Fractions or subfractions with low anti-parasitic activity were excluded from further analysis (IC_50_ > 400 μg/mL).

**Table 2:** Amoebicidal activity against the trophozoite stage of *N. fowleri* (ATCC^®^ 30808™ and ATCC^®^ 30215™) and cytotoxicity against the murine macrophage cell line J774 of ursolic acid (**1**) and derivatives **2**-**11**.

Cpds ^a^	*N. fowleri* ATCC^®^ 30808™	*N. fowleri* ATCC^®^ 30215™	Murine Macrophages	*N. fowleri* ATCC^®^ 30808™	*N. fowleri* ATCC^®^ 30215™
	IC_50_ ^b^ (µM)	IC_50_ ^b^ (µM)	CC_50_ ^c^ (µM)	SI ^d^	SI ^d^
1	141.53 ± 8.17	82.53 ± 8.43	35.93 ± 9.28	0.22	0.43
2	88.70 ± 3.02	84.47 ± 6.31	49.65 ± 5.65	1.12	1.06
7	28.66 ± 1.80	29.69 ± 1.59	>175.19	>6.11	>5.90
8	66.58 ± 9.25	38.71 ± 2.15	84.96 ± 5.00	1.24	2.20
10	73.89 ± 3.22	7.61 ± 1.44	57.80 ± 2.32	0.87	7.60
11	78.55 ± 5.82	34.60 ± 8.86	40.75 ± 4.99	0.84	1.92
M ^e^	38.74 ± 4.23	81.57 ± 7.23	127.89 ± 8.85	3.30	1.57
A ^e^	0.12 ± 0.03	0.16 ± 0.01	>200	>1650.89	>1250

^a^ Cpds: Compounds and reference drugs assayed. ^b^ IC_50_: Concentrations able to inhibit 50% of amoebae, expressed as µM ± standard deviation (SD). ^c^ CC_50_: Concentration able to inhibit 50% of murine macrophages, expressed as µM ± standard deviation (SD). ^d^ SI: Selectivity index (CC_50_/IC_50_). ^e^ M: Miltefosine and A: Amphotericin B were used as positive controls. Inactive compounds **3**–**6** and **9** were excluded (IC_50_ > 200 μM).

**Table 3:** Amoebicidal activity against the cyst stage of *N. fowleri* (ATCC^®^ 30808™ and ATCC^®^ 30215™) and cytotoxicity against the murine macrophage cell line J774 of selected derivatives **7** and **10**.

Cpds ^a^	*N. fowleri* ATCC^®^ 30808^TM^	*N. fowleri* ATCC^®^ 30215^TM^	Murine Macrophages	*N. fowleri* ATCC^®^ 30808^TM^	*N. fowleri* ATCC^®^ 30215^TM^
	IC_50_ ^b^ (µM)	IC_50_ ^b^ (µM)	CC_50_ ^c^ (µM)	SI ^d^	SI ^d^
7	36.65 ± 1.49	28.03 ± 1.59	>175.19	4.78	6.25
10	66.47 ± 20.64	17.14 ± 2.59	57.80 ± 2.32	0.87	3.37
M ^e^	21.52 ± 2.62	37.80 ± 5.60	127.89 ± 8.85	5.94	3.38
A ^f^	0.53 ± 0.03	0.62 ± 0.04	>200	377.36	322.58

^a^ Cpds: Compounds and reference drugs assayed. ^b^ IC_50_: Concentrations able to inhibit 50% of trophozoites, expressed as µM ± standard deviation (SD). ^c^ CC_50_: Concentration able to inhibit 50% of murine macrophages, expressed as µM ± standard deviation (SD). ^d^ SI: Selectivity index (CC_50_/IC_50_). ^e^ M: Miltefosine was used as a positive control. ^f^ A: Amphotericin B was used as a positive control.

## Data Availability

Data are contained within the article and Supplementary Materials.

## Supplementary Materials

The following supporting information can be downloaded at: https://www.mdpi.com/article/10.3390/ijms26189048/s1.
