# Supplementary material for: Mentha rotundifolia, a Source of Amoebicidal Agents Against Naegleria fowleri
Source: Int J Mol Sci. 2025 Sep 17;26(18):9048. doi: 10.3390/ijms26189048 (PMC12470768; doi:10.3390/ijms26189048)
Supplement: Supplementary file 1 [file ijms-26-09048-s001.zip › ijms-3829266-supplementary.pdf]

Electronic Supporting Information

Mentha rotundifolia, a Source of Amoebicidal Agents against *Naegleria fowleri*

Meriam Ben Youssef[1,2,3], Javier Chao-Pellicer[1,4,5], Eduardo Hernández-Álvarez[3], Amani Omrani[1,2,3], Ines Sifaoui[1,4,5], Hichem Sebai[2], Isabel L. Bazzocchi[3], José E. Piñero[1,4,5], Ignacio A. Jiménez[3]*, Jacob Lorenzo-Morales[1,4,5] *

[1]Instituto Universitario de Enfermedades Tropicales y Salud Pública de Canarias, Universidad de La Laguna, 38296 San Cristóbal de La Laguna, Santa Cruz de Tenerife Spain

[2]Laboratory of Functional Physiology and Valorization of Bio-Ressources, Higher Institute of Biotechnology of Beja, University of Jendouba, Beja 382-9000, Tunisia

[3]Instituto Universitario de Bio-Orgánica Antonio González and Departamento de Química Orgánica, Universidad de La Laguna, Avenida Astrofísico Francisco Sánchez 2, 38206 La Laguna, Tenerife, Spain

[4]Departamento de Obstetricia y Ginecología, Pediatría, Medicina Preventiva y Salud Pública, Toxicología, Medicina Legal y Forense y Parasitología, Universidad de La Laguna, C/ Sta. María Soledad s/n, 38200 San Cristóbal de La Laguna, Santa Cruz de Tenerife, Spain

[5]Consorcio Centro de investigación Biomédica en Red, Área de Enfermedades Infecciosas (CIBERINFEC), Instituto de Salud Carlos III, Av. Monforte de Lemos 3-5, Pabellón 11, 28029 Madrid, Spain

* Correspondence: ignadiaz@ull.edu.es, jmlorenz@ull.edu.es

Table of contents

Page S2: **Figures S1 and S2**, ^{1}H NMR and ^{13}C NMR spectra of compound **1**

Page S3: **Figures S3 and S4**, ^{1}H NMR and ^{13}C NMR spectra of compound **2**

Page S4: **Figures S5, S6** and **S7,** ^{1}H NMR, ^{13}C NMR and Mass spectra of compound **3**

Page S5: **Figures S8, S9 and S10**, ^{1}H NMR and ^{13}C NMR and Mass spectra of compound **4**

Page S6: **Figures S11, S12 and S13**, ^{1}H NMR and ^{13}C NMR and Mass spectra of compound **5**

Page S7: **Figures S14, S15 and S16**, ^{1}H NMR and ^{13}C NMR and Mass spectra of compound **6**

Page S8: **Figures S17, S18 and S19**, ^{1}H NMR and ^{13}C NMR and Mass spectra of compound **7**

Page S9: **Figures S20, S21 and S22**, ^{1}H NMR and ^{13}C NMR and Mass spectra of compound **8**

Page S10: **Figures S23, S24 and S25**, ^{1}H NMR and ^{13}C NMR and Mass spectra of compound **9**

Page S11: **Figures S26, S27 and S28**, ^{1}H NMR and ^{13}C NMR and Mass spectra of compound **10**

Page S12: **Figures S29, S30 and S31**, ^{1}H NMR and ^{13}C NMR and Mass spectra of compound **11**

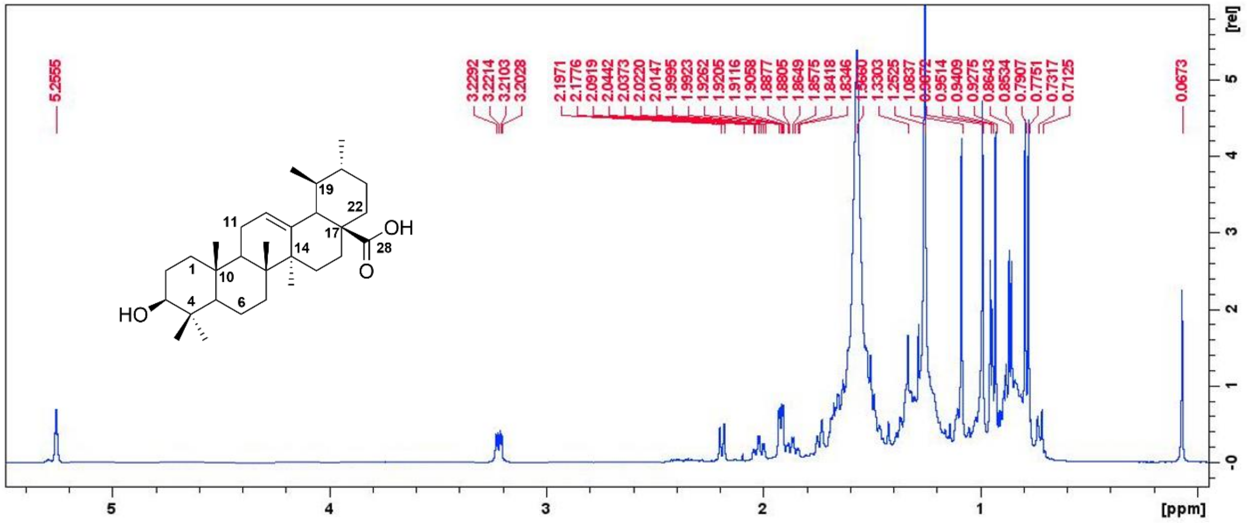

Figure S1. ^{1}H NMR spectrum [600 MHz, $CDCl_3$] of compound **1**.

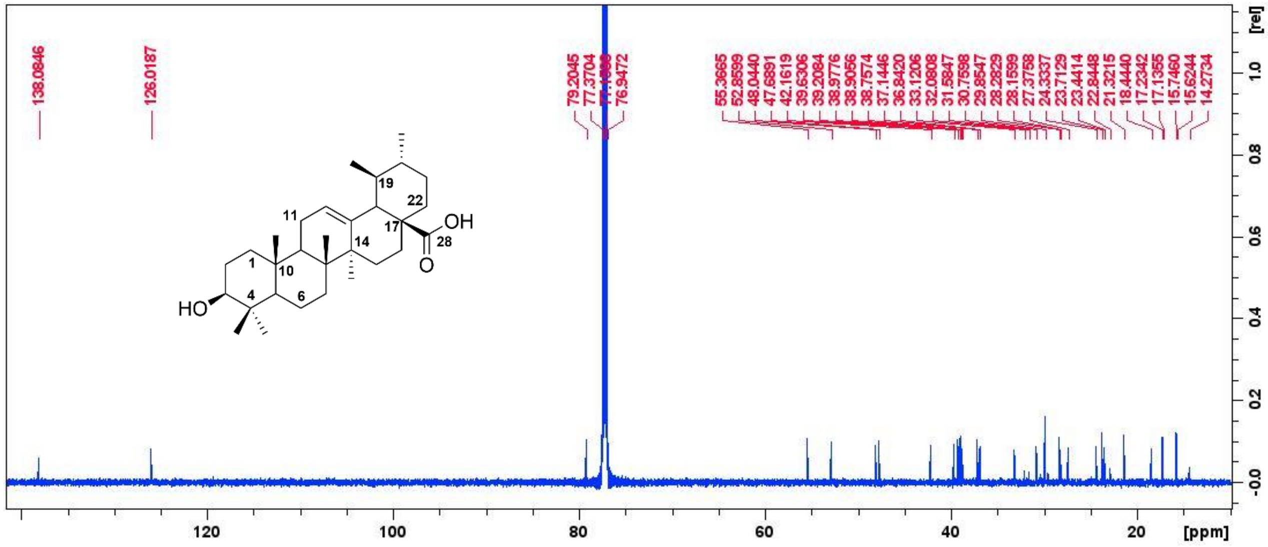

Figure S2. ^{13}C NMR spectrum [150 MHz, $CDCl_3$] of compound **1**.

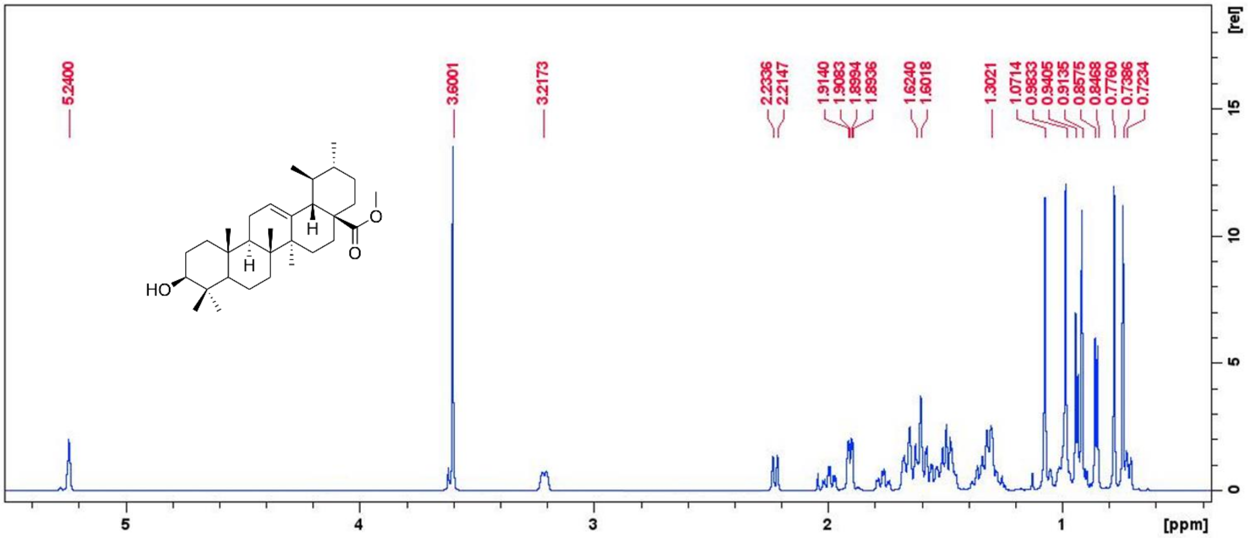

Figure S3. ^{1}H NMR spectrum [600 MHz, $CDCl_3$] of compound **2**.

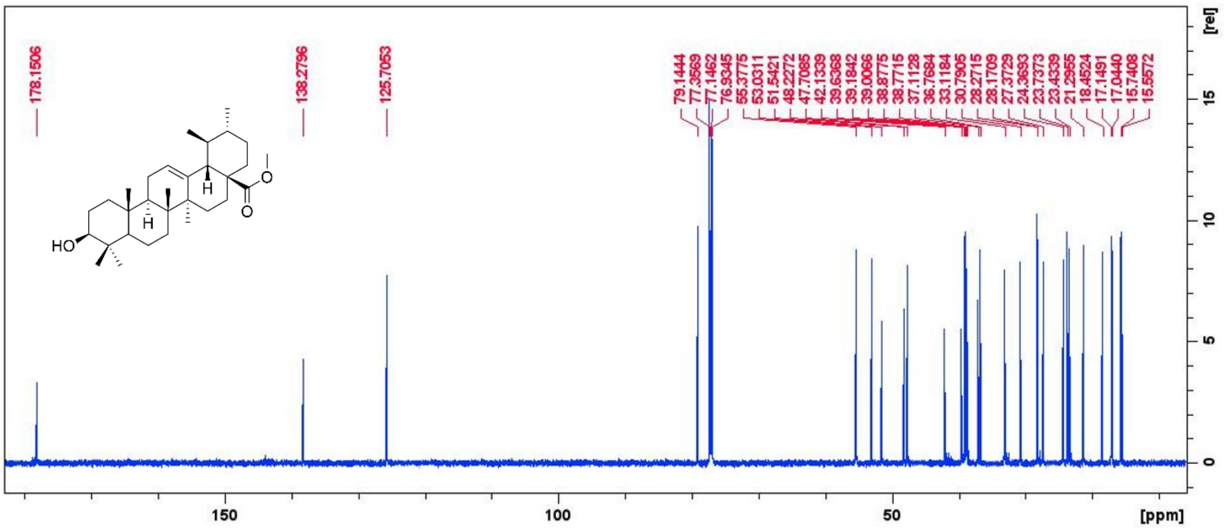

Figure S4. ^{13}C NMR spectrum [150 MHz, $CDCl_3$] of compound **2**.

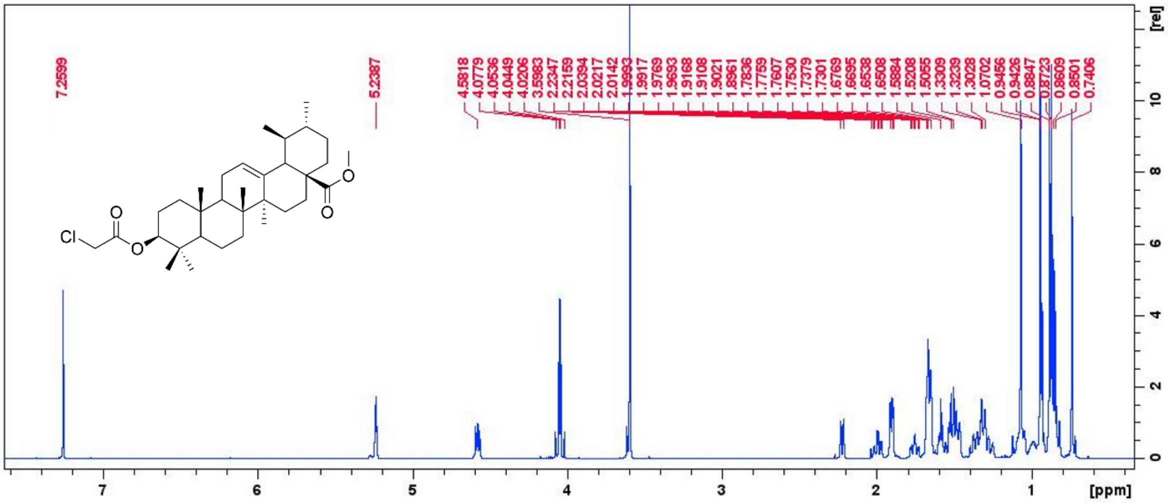

Figure S5. ^{1}H NMR spectrum [600 MHz, $CDCl_3$] of compound **3**.

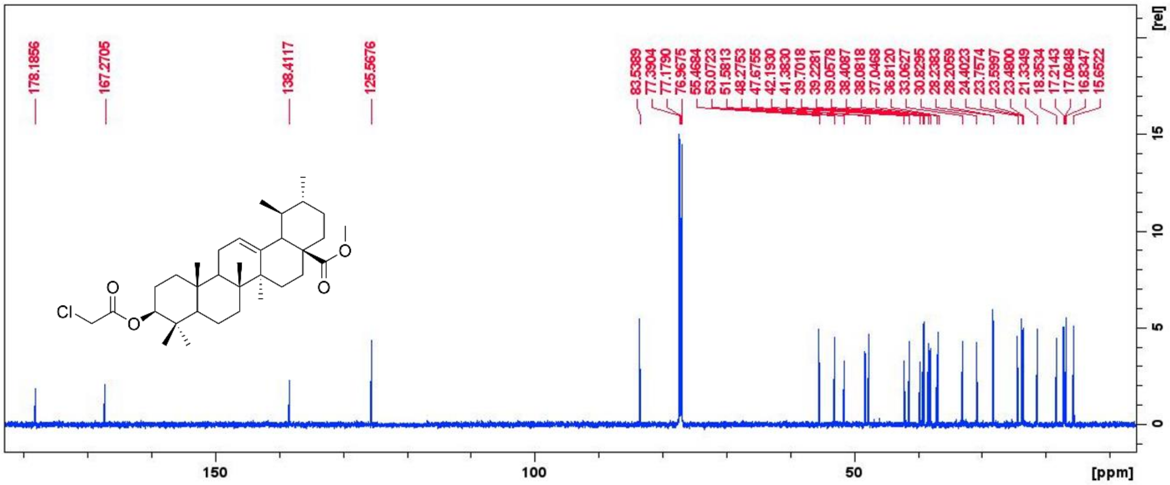

Figure S6. ^{13}C NMR spectrum [150 MHz, solvent $CDCl_3$] of compound **3**.

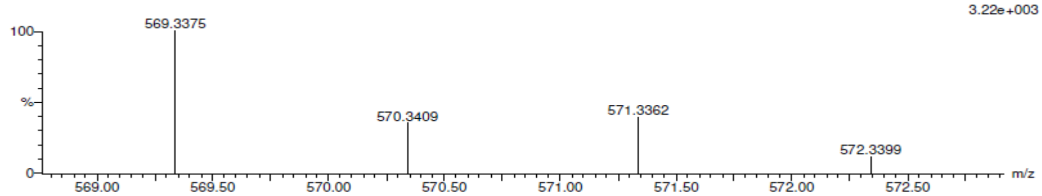

Mass	RA	Calc. Mass	mDa	PPM	DBE	i-FIT	i-FIT (Norm)	Formula
Minimum:	20.00				-1.5			
Maximum:	100.00		5.0	5.0	200.0			
569.3375	100.00	569.3374	0.1	0.2	7.5	24.4	1.0	C33 H51 O4 Na 35Cl
		569.3398	-2.3	-4.0	10.5	24.8	1.4	C35 H50 O4 35Cl
		569.3350	2.5	4.4	4.5	24.3	0.9	C31 H52 O4 Na2 35Cl
570.3409	35.16	---						
571.3362	39.25	571.3368	-0.6	-1.1	10.5	22.1	0.4	C35 H50 O4 37Cl
		571.3344	1.8	3.2	7.5	22.8	1.1	C33 H51 O4 Na 37Cl

Figure S7. Mass spectrum of compound **3**.

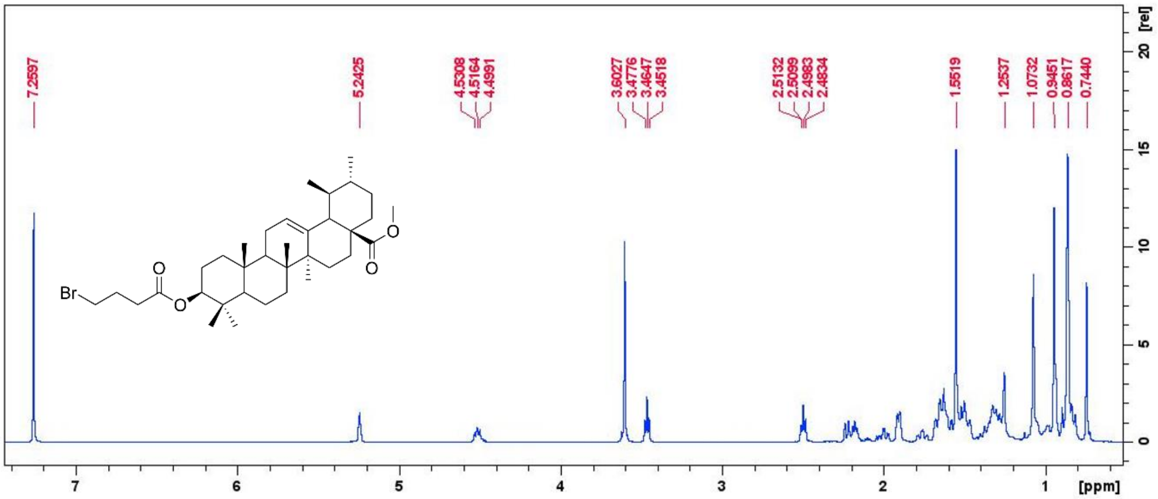

Figure S8. ^{1}H NMR spectrum [500 MHz, $CDCl_3$] of compound **4**.

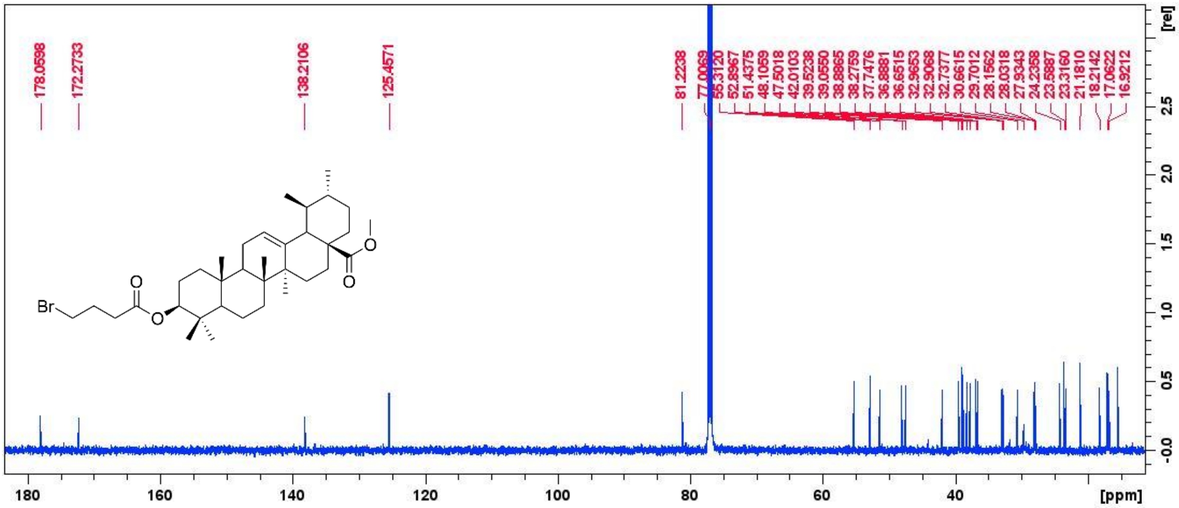

Figure S9. ^{13}C NMR spectrum [125 MHz, $CDCl_3$] of compound **4**.

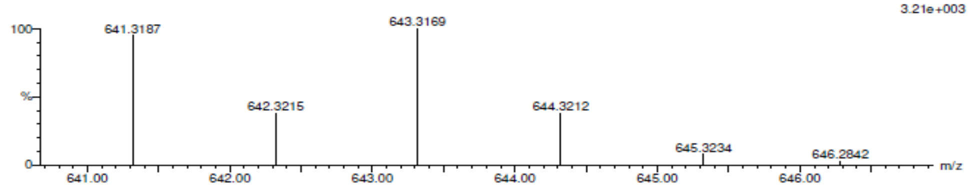

Mass	RA	Calc. Mass	mDa	PPM	DBE	i-FIT	i-FIT (Norm)	Formula
Minimum:	20.00				-1.5			
Maximum:	100.00		5.0	5.0	200.0			
641.3187	95.05	641.3181	0.6	0.9	7.5	28.0	1.1	C35 H55 O4 Na 79Br
		641.3205	-1.8	-2.8	10.5	28.3	1.4	C37 H54 O4 79Br
		641.3157	3.0	4.7	4.5	27.8	0.9	C33 H56 O4 Na2 79Br
642.3215	37.84	---						
643.3169	100.00	643.3161	0.8	1.2	7.5	8.9	0.4	C35 H55 O4 Na 81Br
		643.3185	-1.6	-2.5	10.5	11.4	2.9	C37 H54 O4 81Br
		643.3137	3.2	5.0	4.5	9.8	1.3	C33 H56 O4 Na2 81Br
644.3212	37.71	---						

Figure S10. Mass spectrum of compound **4**.

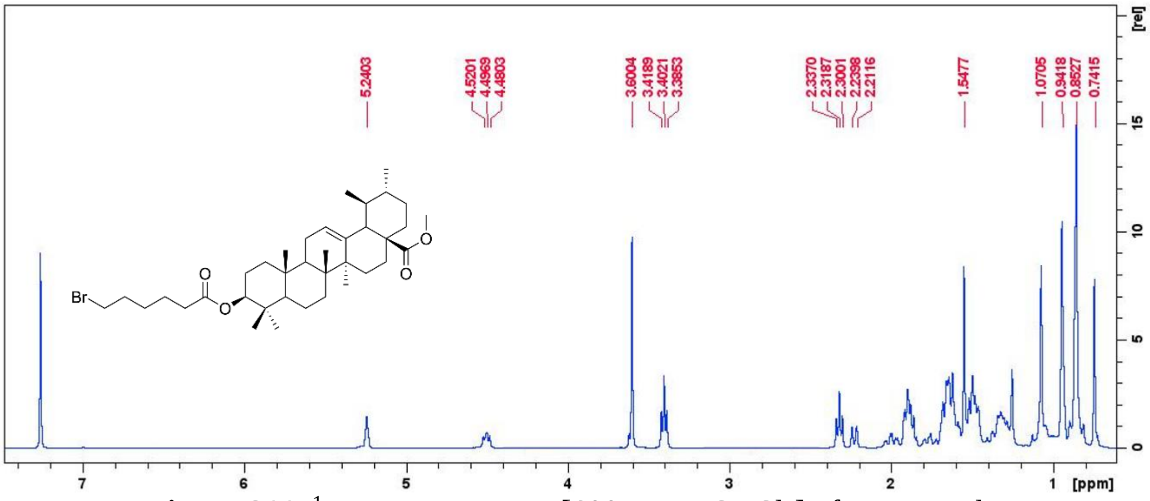

Figure S11. ^{1}H NMR spectrum [600 MHz, $CDCl_3$] of compound **5**.

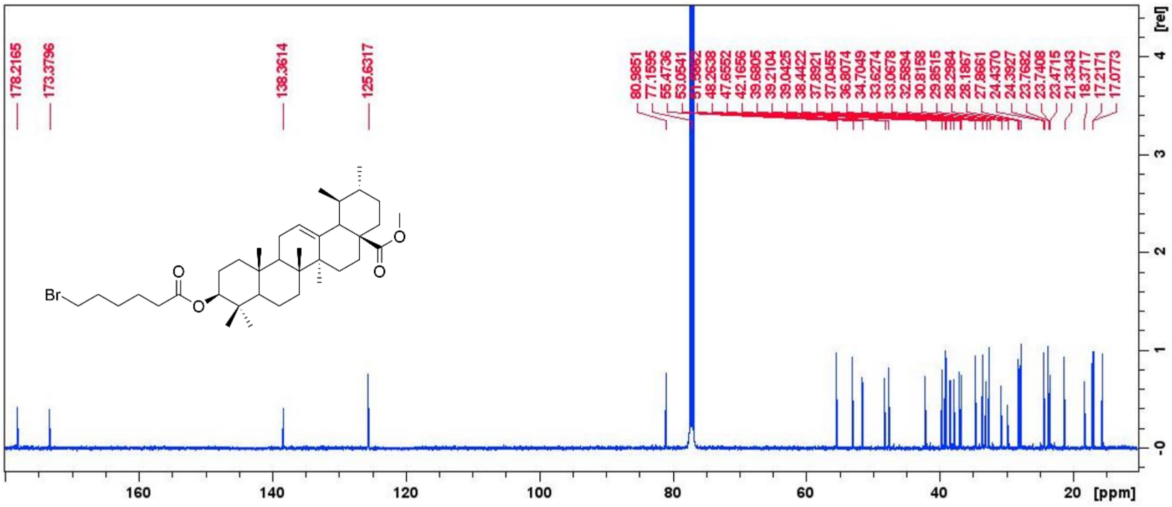

Figure S12. ^{13}C NMR spectrum [125 MHz, solvent $CDCl_3$] of compound **5**.

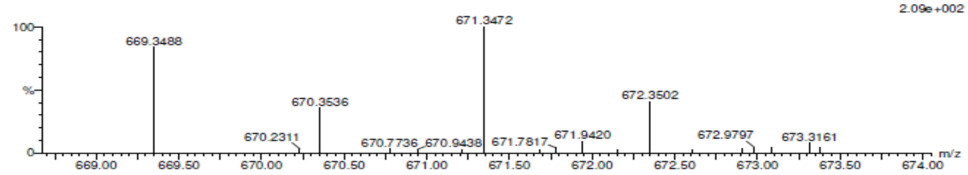

Mass	RA	Calc. Mass	mDa	PPM	DBE	1-FIT	1-FIT (Norm)	Formula
Minimum:	20.00				-1.5			
Maximum:	100.00		5.0	5.0	200.0			
669.3488	83.67	669.3494	-0.6	-0.9	7.5	42.2	1.3	C37 H59 O4 Na 79Br
		669.3470	1.8	2.7	4.5	41.5	0.5	C35 H60 O4 Na2 79Br
		669.3518	-3.0	-4.5	10.5	43.1	2.1	C39 H58 O4 79Br
670.3536	35.49	---						
671.3472	100.00	671.3474	-0.2	-0.3	7.5	51.1	1.3	C37 H59 O4 Na 81Br
		671.3450	2.2	3.3	4.5	50.8	1.0	C35 H60 O4 Na2 81Br
		671.3498	-2.6	-3.9	10.5	51.8	2.0	C39 H58 O4 81Br
		671.3440	3.2	4.8	11.5	51.5	1.7	C40 H57 O2 Na 79Br
672.3502	40.54	---						

Figure S13. Mass spectrum of compound **5**.

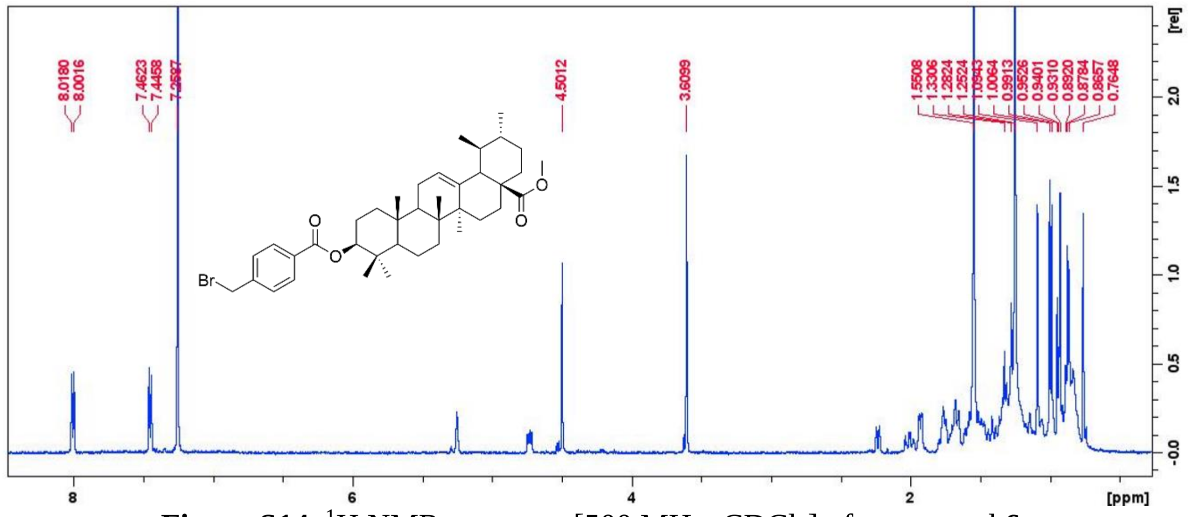

Figure S14. ^{1}H NMR spectrum [500 MHz, $CDCl_3$] of compound **6**.

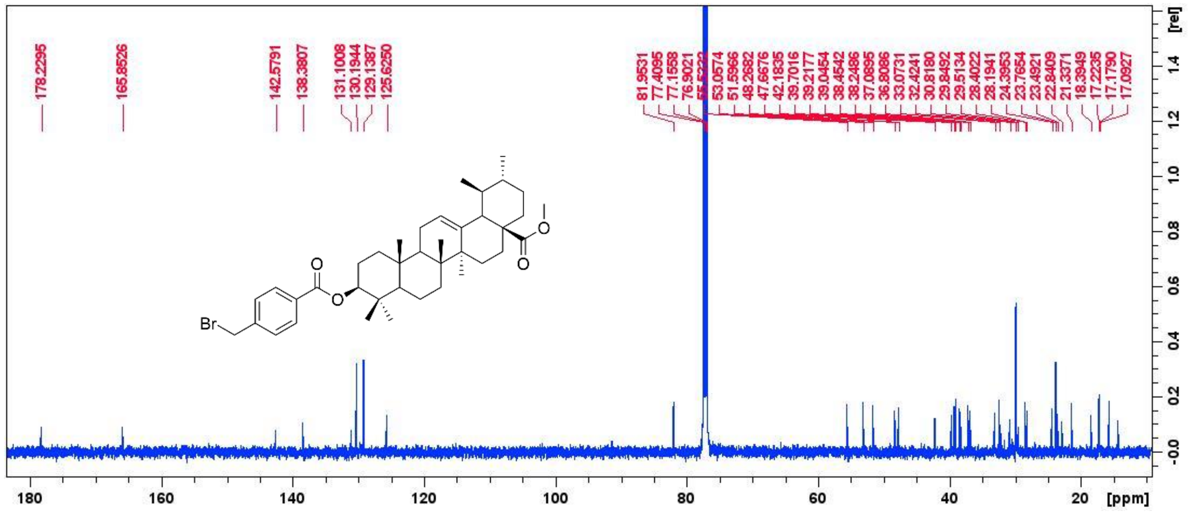

Figure S15. ^{13}C NMR spectrum [125 MHz, $CDCl_3$] of compound **6**.

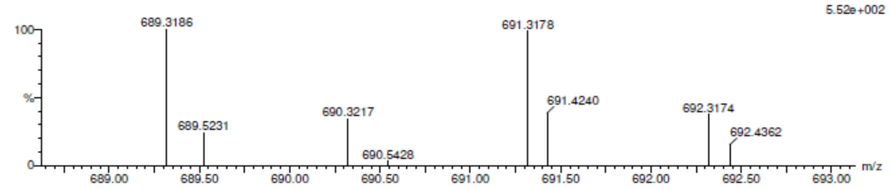

Minimum:	20.00				-1.5			
Maximum:	100.00		5.0	5.0	200.0			
Mass	RA	Calc. Mass	mDa	PPM	DBE	i-FIT	i-FIT (Norm)	Formula
689.3186	100.00	689.3181	0.5	0.7	11.5	47.5	1.0	C39 H55 O4 Na 79Br
		689.3157	2.9	4.2	8.5	47.0	0.5	C37 H56 O4 Na2 79Br
689.5231	23.99	---						
690.3217	33.93	---						
691.3178	98.80	691.3161	1.7	2.5	11.5	40.9	0.0	C39 H55 O4 Na 81Br
691.4240	38.33	691.4228	1.2	1.7	5.5	30.6	1.2	C40 H68 O Na2 81Br
		691.4253	-1.3	-1.9	0.5	29.8	0.4	C36 H70 O4 Na2 79Br
692.3174	37.12	---						

Figure S16. Mass spectrum of compound **6**.

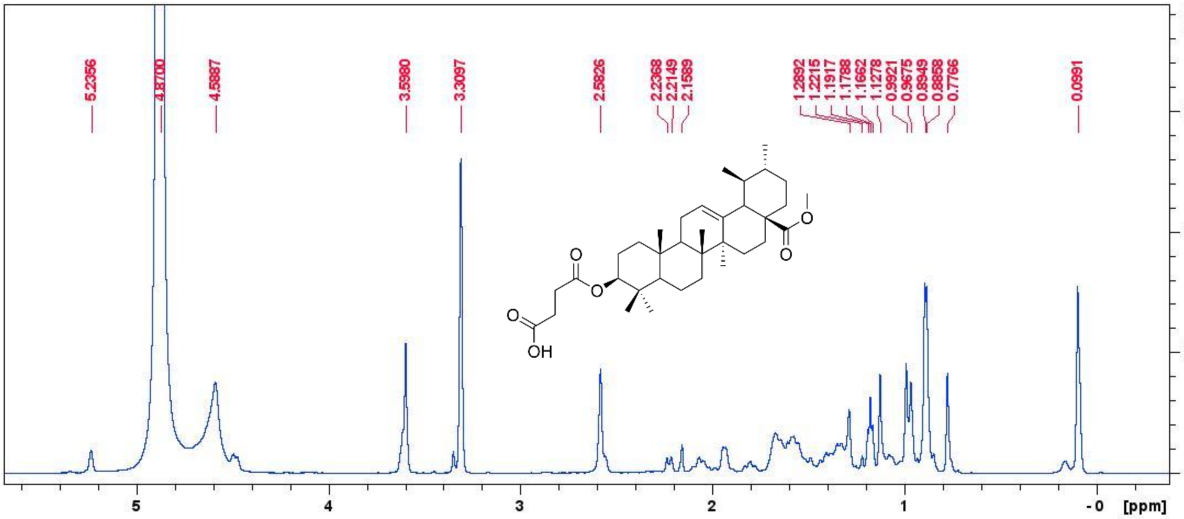

Figure S17. ^{1}H NMR spectrum [500 MHz, CD_3OD] of compound **7**.

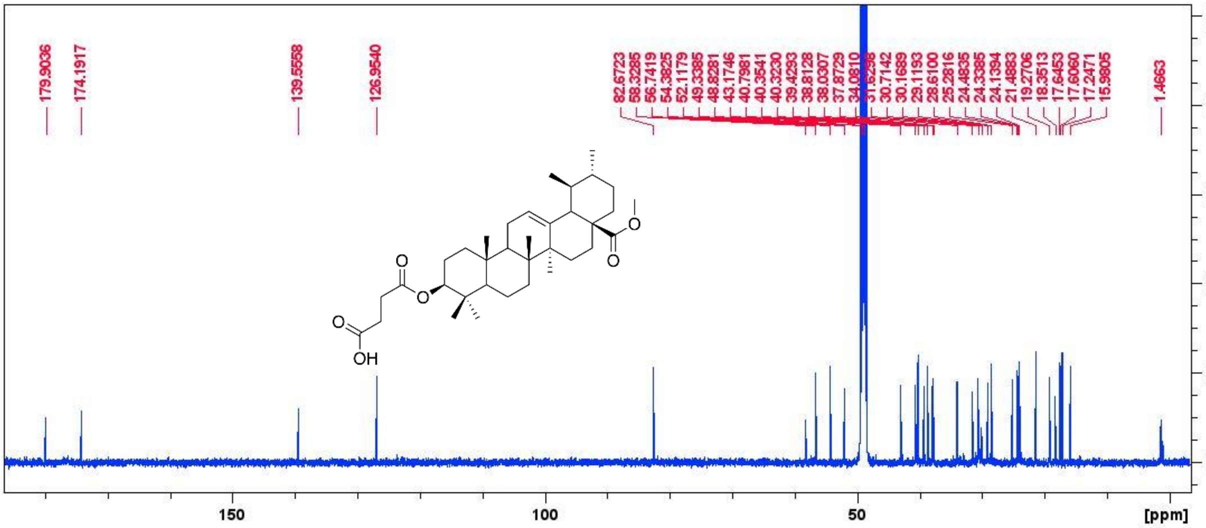

Figure S18. ^{13}C NMR spectrum [125 MHz, CD_3OD] of compound **7**.

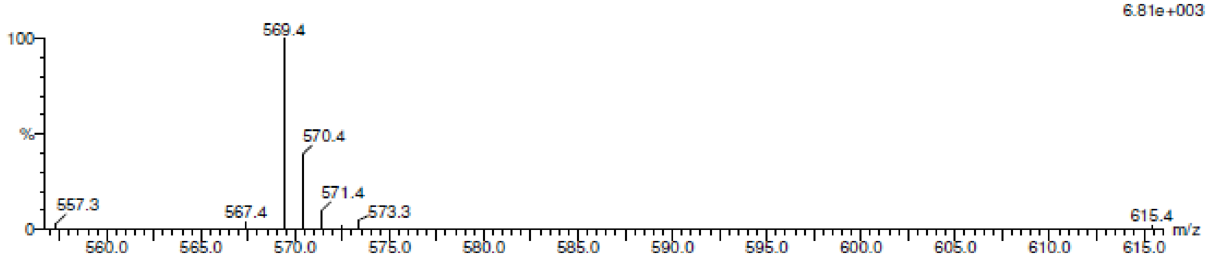

Minimum:	20.00				-1.5			
Maximum:	100.00		5.0	5.0	200.0			
Mass	RA	Calc. Mass	mDa	PPM	DBE	i-FIT	i-FIT (Norm)	Formula
569.3851	100.00	569.3842	0.9	1.6	9.5	9.2	0.0	C35 H53 O6
570.3887	39.08	---						

Figure S19. Mass spectrum of compound **7**.

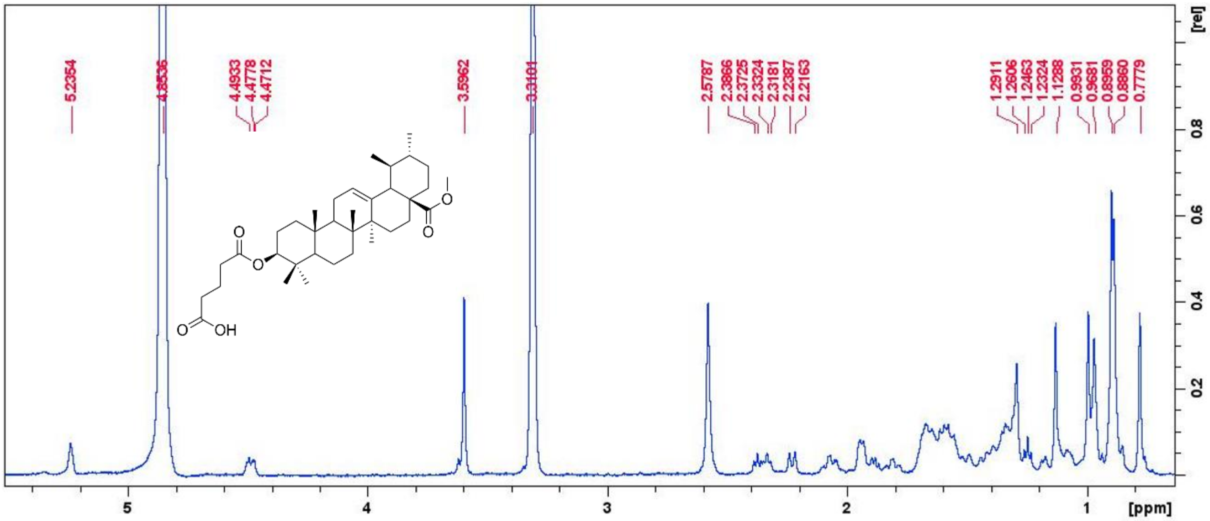

Figure S20. ^{1}H NMR spectrum [500 MHz, CD_3OD] of compound **8**.

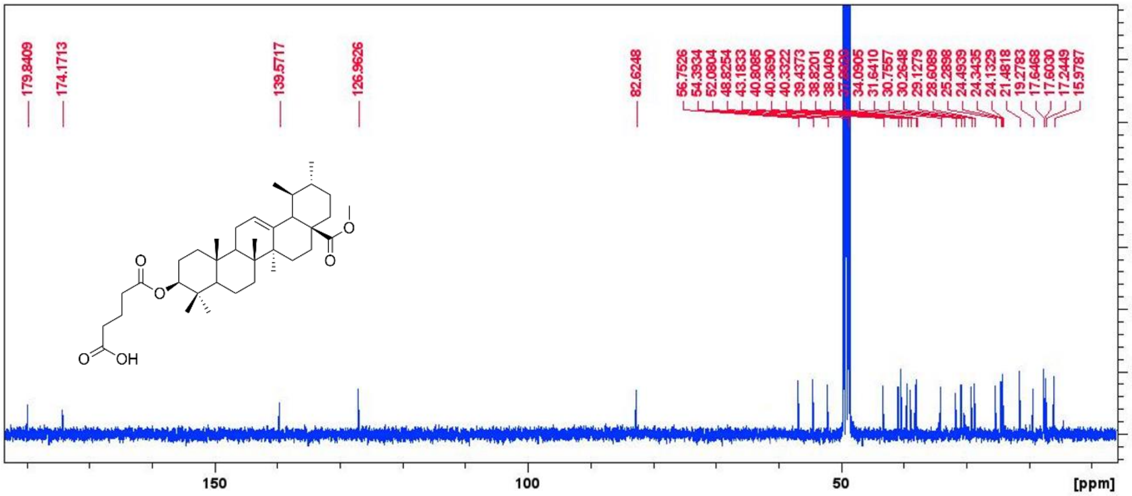

Figure S21. ^{13}C NMR spectrum [125 MHz, CD_3OD] of compound **8**.

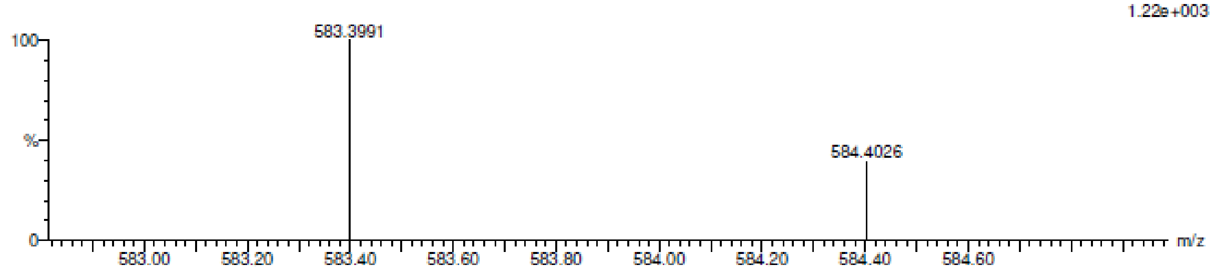

Mass	RA	Calc. Mass	mDa	PPM	DBE	i-FIT	i-FIT (Norm)	Formula
Minimum:	20.00				-1.5			
Maximum:	100.00		5.0	5.0	200.0			
583.3991	100.00	583.3999	-0.8	-1.4	9.5	22.2	0.4	C36 H55 O6
		583.3975	1.6	2.7	6.5	22.8	1.0	C34 H56 O6 Na
584.4026	39.06	---						

Figure S22. Mass spectrum of compound **8**.

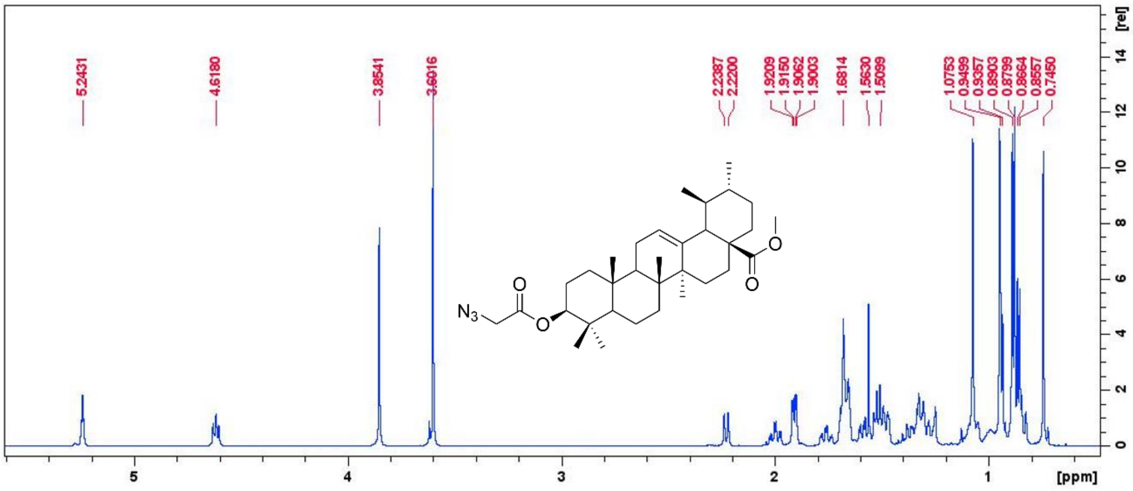

Figure S23. ^{1}H NMR spectrum [600 MHz, $CDCl_3$] of compound **9**.

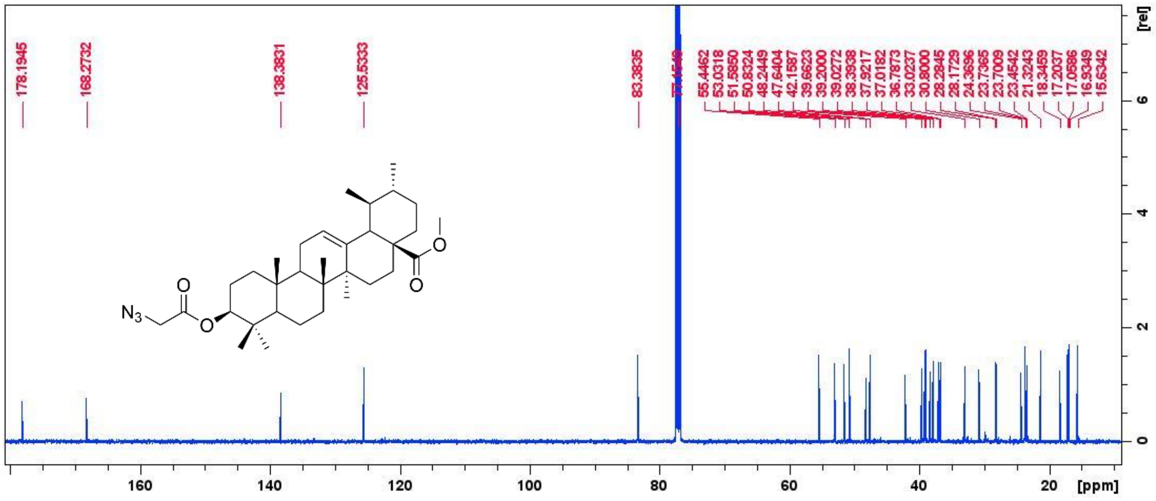

Figure S24. ^{13}C NMR spectrum [125 MHz, $CDCl_3$] of compound **9**.

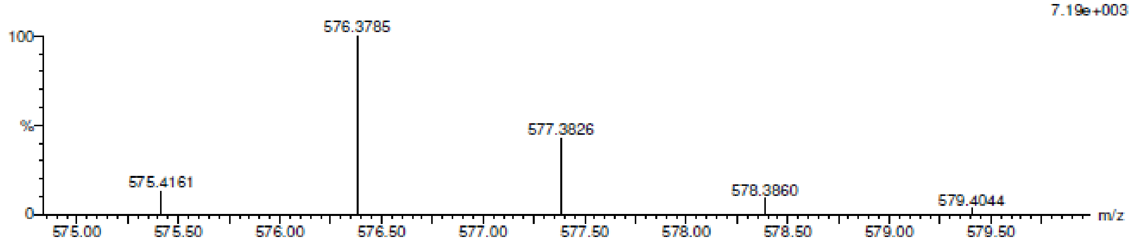

Minimum:	20.00				-1.5			
Maximum:	100.00		5.0	5.0	200.0			
Mass	RA	Calc. Mass	mDa	PPM	DBE	1-FIT	1-FIT (Norm)	Formula
576.3785	100.00	576.3777	0.8	1.4	9.5	11.6	2.7	C33 H51 N3 O4 Na
		576.3801	-1.6	-2.8	12.5	9.0	0.1	C35 H50 N3 O4
577.3826	42.60	---						

Figure S25. Mass spectrum of compound **9**.

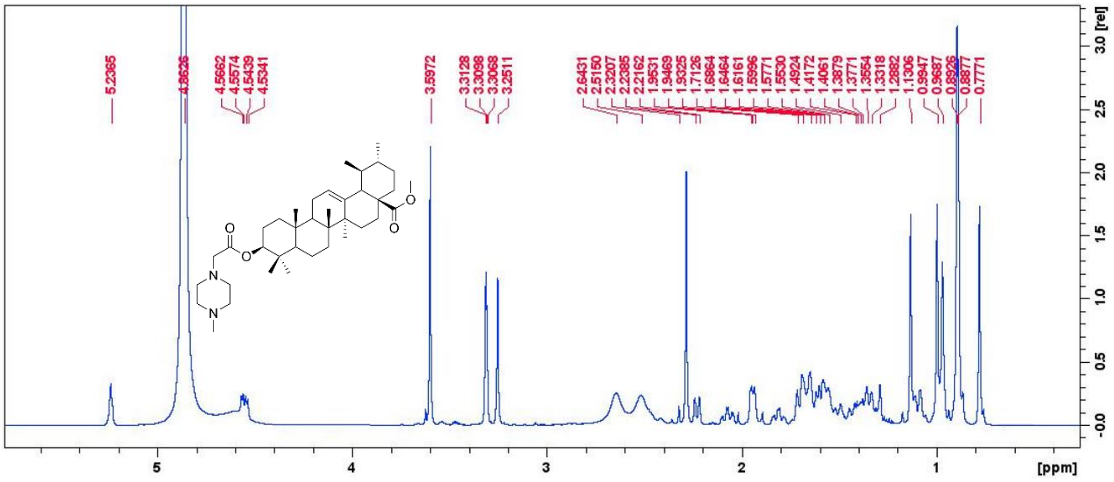

Figure S26. ^{1}H NMR spectrum [500 MHz, CD_3OD] of compound **10**.

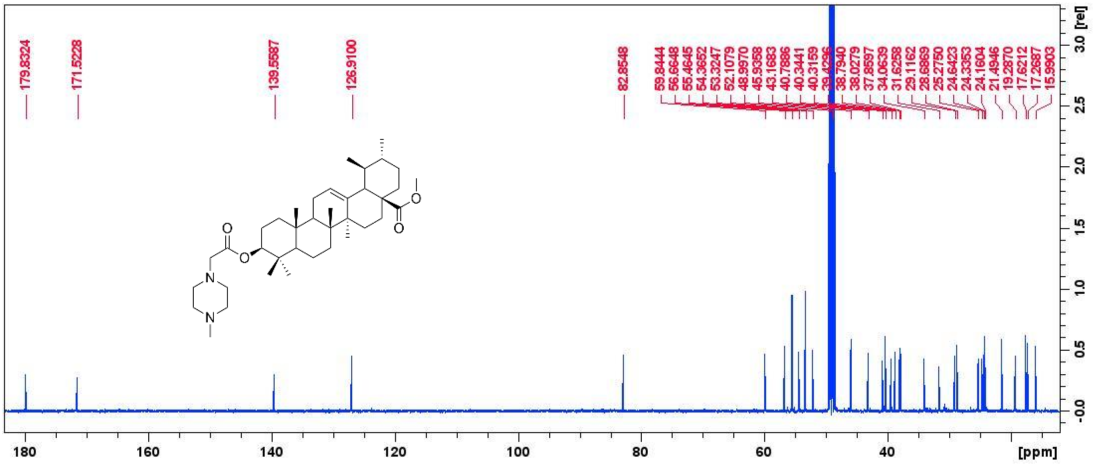

Figure S27. ^{13}C NMR spectrum [125 MHz, CD_3OD] of compound **10**.

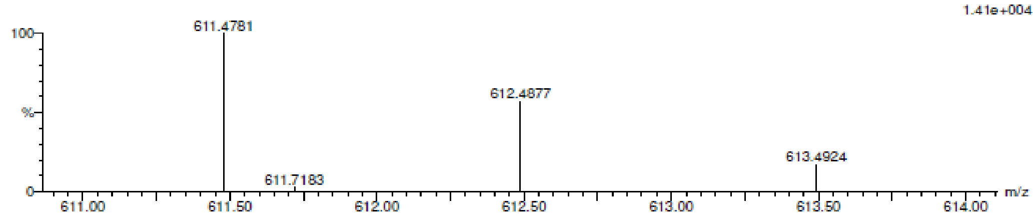

Minimum:	20.00				-1.5			
Maximum:	100.00		5.0	5.0	200.0			

Mass	RA	Calc. Mass	mDa	PPM	DBE	i-FIT	i-FIT (Norm)	Formula
611.4781	100.00	611.4782	-0.1	-0.2	0.5	38.2	2.0	C35 H69 O4 Na 35Cl
		611.4788	-0.7	-1.1	8.5	37.1	0.9	C38 H63 N2 O4
		611.4764	1.7	2.8	5.5	38.1	2.0	C36 H64 N2 O4 Na
		611.4806	-2.5	-4.1	3.5	37.3	1.2	C37 H68 O4 35Cl
612.4877	56.82	---						

Figure S28. Mass spectrum of compound **10**.

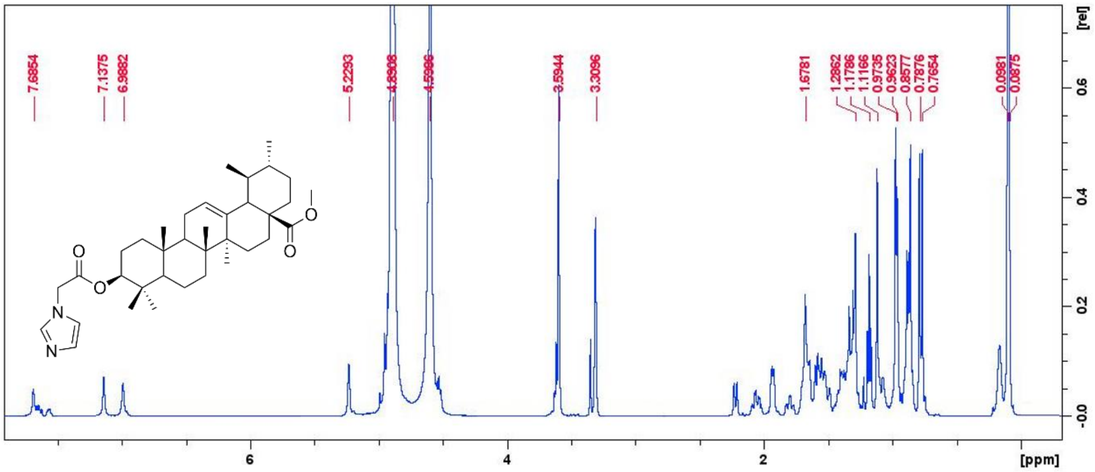

Figure S29. ^{1}H NMR spectrum [500 MHz, CD_3OD] of compound **11**.

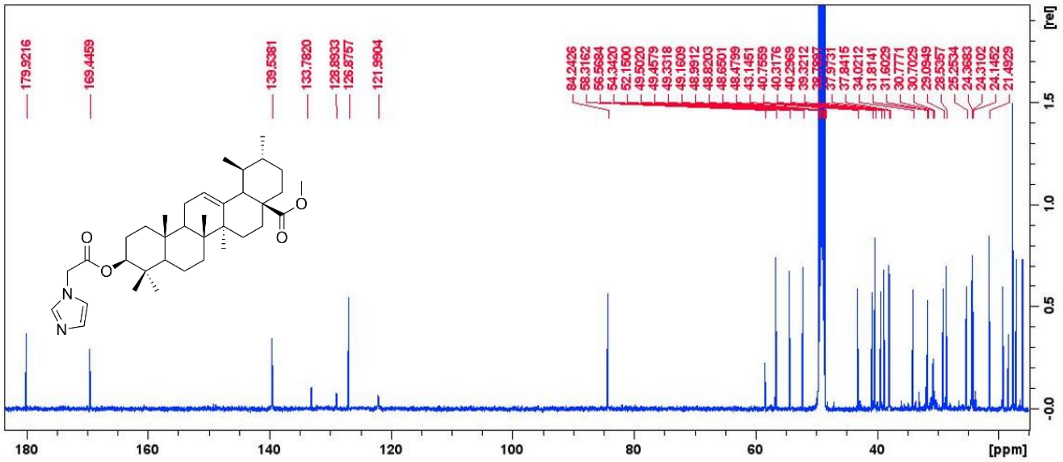

Figure S30. ^{13}C NMR spectrum [125 MHz, CD_3OD] of compound **11**.

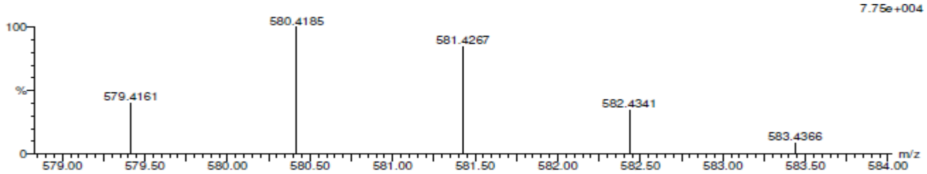

Mass	RA	Calc. Mass	mDa	PPM	DBE	i-FIT	i-FIT (Norm)	Formula
Minimum:	20.00				-1.5			
Maximum:	100.00		5.0	5.0	200.0			
579.4161	40.26	579.4162	-0.1	-0.2	10.5	37.2	1.4	C36 H55 N2 O4
		579.4154	0.7	1.2	8.5	37.0	1.3	C37 H57 O2 Na2
		579.4178	-1.7	-2.9	11.5	36.9	1.1	C39 H56 O2 Na
		579.4138	2.3	4.0	7.5	37.6	1.8	C34 H56 N2 O4 Na
580.4185	100.00	580.4189	-0.4	-0.7	2.5	36.6	1.1	C31 H59 N O7 Na
		580.4165	2.0	3.4	-0.5	37.1	1.6	C29 H60 N O7 Na2
		580.4213	-2.8	-4.8	5.5	36.2	0.7	C33 H58 N O7
581.4267	84.79	581.4270	-0.3	-0.5	3.5	24.4	2.2	C32 H59 N2 O4 Na2
		581.4294	-2.7	-4.6	6.5	22.3	0.1	C34 H58 N2 O4 Na
582.4341	34.57	582.4370	-2.9	-5.0	4.5	33.3	0.0	C33 H60 N O7

Figure S31. Mass spectrum of compound **11**.
